# Identification and Functional Analysis of Two Purple Acid Phosphatases AtPAP17 and AtPAP26 Involved in Salt Tolerance in *Arabidopsis thaliana* Plant

**DOI:** 10.3389/fpls.2020.618716

**Published:** 2021-02-15

**Authors:** Mohammad Ali Abbasi-Vineh, Mohammad Sadegh Sabet, Ghasem Karimzadeh

**Affiliations:** ^1^Department of Agricultural Biotechnology, Faculty of Agriculture, Tarbiat Modares University, Tehran, Iran; ^2^Department of Plant Genetics and Breeding, Faculty of Agriculture, Tarbiat Modares University, Tehran, Iran

**Keywords:** gene expression profiling, SOS pathway, antioxidant activities, overexpressed and mutant plants, APase activity, Pi homeostasis

## Abstract

Tolerance to salinity is a complex genetic trait including numerous physiological processes, such as metabolic pathways and gene networks; thereby, identification of genes indirectly affecting, as well as those directly influencing, is of utmost importance. In this study, we identified and elucidated the functional characterization of *AtPAP17* and *AtPAP26* genes, as two novel purple acid phosphatases associated with high-salt tolerance in NaCl-stressed conditions. Here, the overexpression of both genes enhanced the expression level of *AtSOS1*, *AtSOS2*, *AtSOS3*, *AtHKT1*, *AtVPV1*, and *AtNHX1* genes, involving in the K^+^/Na^+^ homeostasis pathway. The improved expression of the genes led to facilitating intracellular Na^+^ homeostasis and decreasing the ion-specific damages occurred in overexpressed genotypes (OEs). An increase in potassium content and K^+^/Na^+^ ratio was observed in OE17 and OE26 genotypes as well; however, lower content of sodium accumulated in these plants at 150 mM NaCl. The overexpression of these two genes resulted in the upregulation of the activity of the catalase, guaiacol peroxidase, and ascorbate peroxidase. Consequently, the overexpressed plants showed the lower levels of hydrogen peroxide where the lowest amount of lipid peroxidation occurred in these lines. Besides the oxidation resistance, the boost of the osmotic regulation through the increased proline and glycine-betaine coupled with a higher content of pigments and carbohydrates resulted in significantly enhancing biomass production and yield in the OEs under 150 mM NaCl. High-salt stress was also responsible for a sharp induction on the expression of both *PAP17* and *PAP26* genes. Our results support the hypothesis that these two phosphatases are involved in plant responses to salt stress by APase activity and/or non-APase activity thereof. The overexpression of *PAP17* and *PAP26* could result in increasing the intracellular APase activity in both OEs, which exhibited significant increases in the total phosphate and free Pi content compared to the wild-type plants. Opposite results witnessed in mutant genotypes (Mu17, Mu26, and DM), associating with the loss of *AtPAP17* and *AtPAP26* functions, clearly confirmed the role of these two genes in salt tolerance. Hence, these genes can be used as candidate genes in molecular breeding approaches to improve the salinity tolerance of crop plants.

## Introduction

Salinity is one of the major abiotic factors affecting the growth, development, and productivity of agricultural crops (Munns and Gilliham, 2015; Parihar et al., 2015; Zörb et al., 2019). All main processes such as protein synthesis, photosynthesis, and metabolism of lipid and energy are adversely affected by salinity within plants (Woodrow et al., 2017; Zörb et al., 2019). Local salinity may be due to high levels of Cl^–^, SO_4_^2–^, CO_3_^2–^, HCO_3_, Na^+^, K^+^, Mg^+^, or Ca^2+^, but for many plants, Na^+^ and Cl^–^ are the primary cause of ion-specific damage (Hasegawa, 2013). Salt stress causes alterations in plant metabolism, including ionic toxicity, reduction in water potential, and ion imbalances that reduce the uptake of nitrogen, potassium, and phosphorus (Brown et al., 2006; Munns and Tester, 2008; Bargaz et al., 2016; Annunziata et al., 2017). Some studies show that salinity causes phosphorus deficiencies or imbalances in plant cells, and the phosphate deprivation adversely impacts photosynthetic processes (Plaxton and Carswell, 1999; Hammond and White, 2008; Carstensen et al., 2018). Meanwhile, phosphorus plays an important role in plant’s developmental processes at both cellular and whole plant level comprising respiration, photosynthesis, energy metabolism, membrane biosynthesis, regulation of several enzymes involved in protein synthesis, biosynthesis of nucleic acids, signaling pathways, and ion transport (Plaxton, 2004; Tran et al., 2010a; Malhotra et al., 2018).

The application of phosphate fertilizers, at first glance, can lead to improve salt tolerance in plants, which carried out, for example, in bean (Bargaz et al., 2016), eggplant (Elwan, 2010), and spinach (Kaya et al., 2001). By using this approach, the adverse effects of high salinity were alleviated on plant growth. The fact is, however, that the response of plants to the fertilizers depends on various factors such as fertilizer application method, nutrient source, cultivar as well as salt-stress severity (Machado and Serralheiro, 2017). In other words, the optimal fertilizer application rate is a challenging recommendation under salinity (Hatam et al., 2019, 2020). By way of illustration, in plants, such as *Pistacia vera*, supplied with higher concentrations of phosphate, the further phosphate accumulated with toxic effects in tissues of plants could lead to a drop in growth and whole productivity (Shahriaripour et al., 2011). Thus, just a moderate phosphate supply is required to boost salinity tolerance and, more importantly, providing such a precise amount of this nutrient would be problematic for each plant. The strategy used in the supplementary of inorganic fertilizers, most notably, may exacerbate soil salinization, and it is also primarily based on competition between ions; in fact, one ion limits the uptake of another ion (Machado and Serralheiro, 2017; Hatam et al., 2019, 2020). Therefore, we believe plant responses should be very efficient to P deficiency in the salinity-treated cells and accordingly regulate their development and growth.

An alternative approach is that plants themselves develop various adaptation strategies to cope with phosphate deficiency such as further releasing, recycling, and scavenging of inorganic phosphate (Pi) from internal and/or external resources (Goldstein et al., 1988; Duff et al., 1994; Del Pozo et al., 1999). The hydrolysis of the phosphate from a wide variety of anhydrides and phosphate esters is catalyzed by purple acid phosphatases (PAPs), a group of acid phosphatases (APase), in plants (Veljanovski et al., 2006; Plaxton and Tran, 2011).

However, a thorough understanding of the physiological functions of different classes of PAPs has not been accomplished in various conditions (Liao et al., 2003). The PAP family comprises 29 members in *Arabidopsis thaliana* (AtPAPs) genome, among which *AtPAP17* and *AtPAP26* play a critical role in the mobilization and utilization of intracellular or extracellular Pi (Wang et al., 2011, 2014; Robinson et al., 2012; Farhadi et al., 2020). Interestingly, *AtPAP17* (*AtACP5*) is not only induced by Pi deprivation but also its expression level is increased by high salt stress and oxidative stress (Del Pozo et al., 1999). When *Arabidopsis* plants were exposed to salt, nitrogen starvation, cold, Pi deprivation, and sulfur starvation, the expression level of *AtPAP26* gene is only induced by no-Pi and salt treatments (Lohrasebi et al., 2007).

Furthermore, the expression patterns of these genes with phosphatase and peroxidase activities in different environmental conditions support the hypothetical multifunctional activities of these two PAP enzymes (Del Pozo et al., 1999; Veljanovski et al., 2006). PP2A phosphatases, for more example, play a critical role in the regulatory reactive oxygen species (ROS) signaling networks in plants, which control metabolic changes, and contribute to transcriptional and post-translational regulation of antioxidant enzymes and pro-oxidant (Rahikainen et al., 2016). Therefore, it is proposed that *AtPAP17* and *AtPAP26* genes could play a role in the signaling of activities of ROS scavenging enzymes in salt-stress conditions.

Consequently, since nearly all cellular activities in eukaryotes are modulated by reversible phosphorylation, phosphatase functions embrace the whole range of cellular and developmental processes under various conditions (Luan, 1998; DeLong, 2006; Singh et al., 2010). In fact, several studies revealed that genes encoding protein phosphatases are critical components in signaling pathways responsible for ion-channel regulation in guard cells, abscisic acid (ABA) functions, vacuolar channel management, developmental processes, light-responsive transcription, regulation of abiotic stress responses, and so on (Sopory and Munshi, 1998; Luan, 2003; Schweighofer et al., 2004; DeLong, 2006; Singh et al., 2010). Therefore, *AtPAP17* and *AtPAP26* genes with APase activity genes and non-APase activity involved in adaptation responses more likely to take responsibility under salinity stress conditions.

The ultimate aim of this salinity tolerance research was to boost the ability of plants to reduce the negative influence of the stress in saline environments, to maintain growth and productivity. Having ensured about effects of *AtPAP17* and *AtPAP26* genes in response to salt stress, followed by understanding how these multifunctional genes respond to salt stress could help us to develop a better strategy for improving salt stress tolerance in crop plants. For this purpose, we evaluated the molecular, physiological, biochemical, and morphological effects of the overexpression and knockout mutation of *AtPAP17* and *AtPAP26* genes in transgenic and mutant plants on Na^+^ management compare to the wild-type ones.

## Materials and Methods

### Plant Materials and Culture Conditions

Besides *A. thaliana* ecotype Columbia-0 (Col-0), as wild-type plants, five other genotypes were used for all experiments in the current study: lines of atpap17 mutant (Mu17), atpap26 mutant (Mu26), atpap17/26 double mutant (DM), AtPAP17 overexpressing (OE17), and AtPAP26 overexpressing (OE26) (Farhadi et al., 2020).

Seeds were surface sterilized and stratified for 48 h at 4°C, and pre-germinated on solid MS medium, pH 5.8, containing 1% sucrose, and 0.7% agar for 11 days. Before exposing to the treatments, 11-day old seedlings were transferred into 15 ml of half-strength liquid MS medium supplemented with 1.25 mM KH_2_PO_4_. All samples were incubated for 3 days on an orbital shaker at 90 rpm to achieve identical nutritional states. Subsequently, the 14-day old seedlings were subjected to 0, 50, 100, and 150 mM NaCl on half-strength liquid MS medium for 12 days. The culture medium was refreshed every 72 h for each sample. All genotypes were grown at 25°C under fluorescent light (1000 Lux) with a 16:8 h light/dark photoperiod in a growth chamber. Then, the shoot and root tissues of 26-day-old plantlets were collected for further analysis. The seeds of all genotypes were planted in peat:perlite:vermiculite in the ratio of 1:1:1 (*v*/*v*/*v*). After seeds stratification, the plants were transferred into the growth chamber under the mentioned conditions. Sub-irrigation was performed every 48 h with a similar Hoagland’s solution containing 1.25 mM KH_2_PO_4_ for 28 days. Subsequently, the seedlings were subjected to salt stress by applying 50, 100, and 150 mM NaCl with the same Hoagland’s solution, containing 1.25 mMKH_2_PO_4_ for 16 days. The control plants were grown without the addition of NaCl.

### Measurement of Parameters

All samples (shoots and roots) were oven-dried for 48 h at 70°C. The amounts of photosynthetic leaf pigments (chlorophyll *a*, *b*, and carotenoid) were estimated, using acetone (80%), proposed by Arnon (1967). Total available carbohydrates content, water-soluble, and insoluble carbohydrate were assayed using the Sheligl (1986) method. Total protein concentration was determined according to Bradford (1976), using bovine serum albumin (BSA) as a standard. The content of malondialdehyde (MDA) was measured through the thiobarbituric acid (TBA) reaction as described by Hodges et al. (1999). The capacity of H_2_O_2_ accumulation in leaves was obtained after H_2_O_2_ reaction with potassium iodide (KI), following the method proposed by Alexieva et al. (2001). Guaiacol peroxidase (POD) activity was analyzed according to Malik and Singh (1980), catalase (CAT) activity was measured according to the method of Aebi (1974) and ascorbate peroxidase (APX) activity was assayed as described by Nakano and Asada (1981). Measurement of total anthocyanin and flavonoid contents were quantified based on the Krizek et al. (1998) method. Proline content was obtained by following the method of Bates et al. (1973) and glycine betaine was performed according to Grieve and Grattan (1983). The content of Na^+^ and K^+^ were measured by using the flame spectrometry according to Hamada and El-Enany (1994). The spectrophotometric measurement of total and organic phosphate content was performed by following the method described by Ames (1966). Pi released by APase activity was evaluated using *p*-nitrophenyl phosphate (*p*-NPP) as a generic phosphatase substrate according to Naseri et al. (2004). The yield and yield components of treated pot-grown plants were estimated by evaluating the flowering percent, pods number per plant, seeds number per each pod, and l000-seed weight obtained during the salt stress period. Finally, the total seed yield (total seed yield obtained from pods N. × seeds N. per each pod × l000-seed weight) was also calculated for all plants.

### cDNA Synthesis and Gene Expression Analysis

Total RNA was extracted from shoot and root tissues, using RNX reagent (Cinaclon BioScience Co., Iran) according to the manufacturer’s instruction. The RNA samples were further treated with RNase A-free DNase (Roche, Basel, Switzerland) according to the supplier instruction to eliminate genomic DNA contamination. For cDNA synthesis, 1 μg of DNase-treated RNA was reverse transcribed by M-MLV reverse transcriptase (Fermentas, Lithuania) as stated by the manufacturing instructions. A series of semi-quantitative RT-PCR was conducted to evaluate the level of relative gene transcript variants (Zamani et al., 2012; Sabet et al., 2018), and their band intensity in agarose gel was quantified by TotalLab software (Phoretix International, New Castle, United Kingdom). The RT-PCR conditions were as follows: 94°C for 5 min; 35 cycles of 94°C for 1 min, specific annealing temperature for each primer pair for 1 min and 72°C for 1 min; and a final extension step at 72°C for 10 min. The α-*tubulin* was used as the internal control gene to normalize the expression level in the semi-quantitative RT-PCR. the primers of *SOS3*, *SOS2*, *HKT1*, *NHX1*, *AVP1*, *SOS1, PAP17*, *PAP26*, and α*-tubulin* genes in *A. thaliana* were designed by Oligo 7 software (Table 1).

**TABLE 1:** The sequences of each primer pair used to PCR.

Gene name	Accession number	Primer sequence
α*-Tubulin*	AT4G14960	5′-GCTTTCAACACCTTCTTCAG-3′
		5′-GAATAGTTCGCTTGGTCTT-3′
*AtSOS1*	AT2G01980	5′-TCTCTTCGTCGGAATGTCTCTGG-3′
		5′-TAAGCCAGTCAGCAGGTCCTAGC-3′
*AtSOS2*	AT5G35410	5′-AACGGATCTGCACGGACGTTCG-3′
		5′AGCTAACTGTCCGGCCTTGATCG-3′
*AtSOS3*	AT5G24270	5′-CCGAGCTTCTTGCATCCGTCAC-3′
		5′-GCACAGTACACAAGGCAAGTCCA-3′
*AtNHX1*	AT5G27150	5′-AGCAAGGGACCGTACACGTCTC-3′
		5′-TCAGACCGGTTGCAGCACCAAGC-3′
*AtHKT1*	AT4G10310	5′-GAGGATCGCTGTGACGTTGAGACTG-3′
		5′-GCAGCCACCATCGCTGATGTC-3′
*AtAVP1*	AT1G15690	5′-TGCATTCAGGTCTGGTGCTGTGATG-3′
		5-GCTCCAATAGCAGCAGTGGTGTTT-3′
*AtPAP17*	AT3G17790	5′-CGAGTCTGAGTTTGCTGTTGT-3′
		5′-ACATAAGAGTTGCGAGATGGAAC-3′
*AtPAP26*	AT5G34850	5′-ATAGGCGATATGGGTCAGACATTC-3′
		5′-CAGCGTACCAAAGAGGACTGCTAC-3′

### Statistical Analysis

Analysis of variance (ANOVA) was conducted based on a randomized complete block design (RCBD) with three replicates (45 seedlings *in vitro* and 15 seedlings *in vivo* studied in each replicate/treatment) to detect the statistically significant effects of different treatments. The significant mean differences were evaluated by Fisher’s least significant difference (LSD) or the Student’s *t*-test. Likewise, the regression slope and the correlation coefficient levels of significance (for the relationship between the variables) are represented by ^∗^ (*P* ≤ 0.05), ^∗∗^ (*P* ≤ 0.01), and ns (non-significant; *P* > 0.05). To provide a global overview of the impacts of AtPAP17 and AtPAP26 on all measured parameters, hierarchical clustering analysis of variance normalized data was performed as a heat map, using http://www.metaboanalyst.ca/faces/upload/StatUploadView.xhtml. A heat map of Pearson’s correlation selected correlation and principal component analysis were also carried out through the website. Other statistical analyses were performed using SAS V. 9.2 and SPSS V. 22 software at *P* < 0.05.

## Results

### AtPAP17 and AtPAP26 Are Involved in Salt Tolerance

#### Biomass Assessments

To investigate the final effect of salt stress and the response of different genotypes, biomass production was measured, and therein, salt stress caused a strong reduction of biomass in all genotypes studied (Table 2). Nevertheless, the decline was highly dependent on the level of NaCl concentrations and the genotypes as well. Our results showed that salinity inhibited the shoots’ growth more than that of the roots in all genotypes (*t* < 0.01). With NaCl concentration increasing in the growth medium (50 to 150 mM), overexpressed and mutant plants showed the lowest and highest reduction in fresh and dry weight of shoots and roots compared to the wild-type ones, respectively (Table 2). In addition, comparing the genotypes in 100 mM NaCl, knockout mutation of *PAP17* and *PAP26* genes led to a decrease in the fresh and dry weight of shoots and roots in mutant plants (Mu17, Mu26, and DM) compared to those in WT plants (Table 2). However, a significant increase of the parameters was presented in each overexpressed genotype (OE17 and OE26) in 150 mM NaCl comparing with control plants (Table 2).

**TABLE 2:** Means (±SE) of the fresh and dry weight, total carbohydrate, and photosynthetic pigments contents in genotypes subjected to NaCl concentrations.

	Genotype	Fresh weight (mg plant^–1^)		Dry weight (mg plant^–1^)		Total carbohydrate (mg g^–1^DW plant^–1^)	Photosynthetic pigments (mg g^–1^FW plant^–1^)		
		Shoot	Root	Shoot	Root		Chlorophyll *a*	Chlorophyll *b*	Carotenoid
0 mM	WT	36.67 ± 2.77^c^	9.07 ± 0.16^b^	30.22 ± 1.21^c^	7.00 ± 1.11^b^	24.31 ± 0.46^c^	1.79 ± 0.08^ab^	1.05 ± 0.48^a^	0.63 ± 0.04^cb^
NaCl	Mu17	30.16 ± 2.86^d^	11.70 ± 0.82^a^	22.91 ± 2.12^d^	9.16 ± 0.73^a^	25.18 ± 1.62^c^	1.64 ± 0.11^b^	1.24 ± 0.03^a^	0.64 ± 0.08^b^
	Mu26	32.78 ± 1.68^d^	11.87 ± 0.61^a^	26.18 ± 1.18^d^	9.38 ± 0.51^a^	12.58 ± 0.61^e^	1.59 ± 0.07^b^	1.19 ± 0.11^a^	0.57 ± 0.02^cb^
	DM	42.89 ± 1.93^b^	12.15 ± 0.27^a^	36.12 ± 1.74^ab^	9.60 ± 0.23^a^	20.78 ± 0.50^d^	1.61 ± 0.21^b^	1.15 ± 0.06^a^	0.55 ± 0.04^c^
	OE17	46.91 ± 1.28^a^	11.51 ± 0.64^a^	38.72 ± 2.03^a^	8.87 ± 0.51^a^	42.81 ± 1.47^a^	1.98 ± 0.15^a^	1.43 ± 0.13^a^	0.79 ± 0.03^a^
	OE26	42.00 ± 0.56^b^	7.95 ± 0.35^c^	34.86 ± 1.44^b^	5.95 ± 0.44^b^	29.17 ± 0.57^b^	2.01 ± 0.24^a^	1.37 ± 0.17^a^	0.73 ± 0.04^a^
50 mM	WT	24.20 ± 1.70^c^	10.31 ± 0.56^cb^	18.00 ± 2.01^c^	8.08 ± 0.52^a^	22.55 ± 1.66^b^	1.23 ± 0.82^b^	1.46 ± 0.05^b^	0.54 ± 0.06^b^
NaCl	Mu17	21.16 ± 1.34^d^	13.78 ± 0.47^a^	14.58 ± 0.61^d^	8.58 ± 0.25^a^	14.04 ± 0.77^d^	0.50 ± 0.03^d^	0.61 ± 0.04^d^	0.46 ± 0.02^c^
	Mu26	19.43 ± 1.70^d^	10.52 ± 0.52^b^	11.76 ± 0.67^e^	8.09 ± 0.51^a^	17.47 ± 0.44^c^	1.03 ± 0.20^cb^	0.77 ± 0.04^d^	0.43 ± 0.02^c^
	DM	35.00 ± 0.80^b^	8.73 ± 0.67^d^	27.85 ± 0.68^a^	6.62 ± 0.53^b^	17.24 ± 1.11^c^	0.98 ± 0.14^c^	1.02 ± 0.18^c^	0.34 ± 0.04^d^
	OE17	43.02 ± 1.82^a^	9.96 ± 0.98^cb^	27.35 ± 1.71^a^	7.68 ± 0.50^ab^	29.96 ± 0.63^a^	1.86 ± 0.06^a^	1.91 ± 0.11^a^	0.78 ± 0.06^a^
	OE26	36.42 ± 1.70^b^	9.61 ± 0.63^c^	21.68 ± 0.93^b^	6.67 ± 0.81^b^	23.03 ± 0.86^b^	1.65 ± 0.16^a^	1.47 ± 0.09^b^	0.61 ± 0.02^b^
100 mM	WT	18.36 ± 0.78^b^	9.07 ± 0.26^a^	12.94 ± 0.70^c^	7.12 ± 0.75^a^	18.59 ± 0.53^b^	0.51 ± 0.04^c^	0.84 ± 0.13^c^	0.44 ± 0.07^b^
NaCl	Mu17	14.20 ± 1.32^c^	6.21 ± 0.78^b^	7.94 ± 0.42^d^	4.57 ± 0.53^b^	11.44 ± 1.30^c^	0.30 ± 0.02^d^	0.48 ± 0.04^e^	0.16 ± 0.01^c^
	Mu26	15.21 ± 0.57^c^	4.98 ± 0.99^b^	8.00 ± 0.18^d^	3.40 ± 0.63^c^	9.17 ± 0.92^d^	0.45 ± 0.04^cd^	0.64 ± 0.07^d^	0.39 ± 0.04^b^
	DM	13.47 ± 0.37^c^	2.66 ± 0.28^c^	8.48 ± 1.01^d^	2.37 ± 0.81^d^	12.13 ± 1.01^c^	0.49 ± 0.03^c^	0.67 ± 0.05^d^	0.24 ± 0.01^c^
	OE17	32.42 ± 0.51^a^	8.90 ± 0.52^a^	23.90 ± 0.54^a^	6.87 ± 0.39^a^	33.85 ± 1.73^a^	1.05 ± 0.15^a^	1.29 ± 0.12^a^	0.61 ± 0.03^a^
	OE26	31.46 ± 3.62^a^	8.64 ± 1.34^a^	21.37 ± 1.21^b^	6.40 ± 0.31^a^	11.76 ± 1.01^c^	0.88 ± 0.04^b^	1.07 ± 0.05^b^	0.58 ± 0.00^a^
150 mM	WT	13.60 ± 0.76^c^	3.98 ± 0.52^b^	8.26 ± 1.08^c^	2.77 ± 0.42^b^	9.45 ± 0.33^c^	0.41 ± 0.03^b^	0.57 ± 0.09^c^	0.29 ± 0.04^b^
NaCl	Mu17	7.51 ± 0.23^e^	1.98 ± 0.06^c^	5.63 ± 0.29^d^	0.75 ± 0.06^d^	3.96 ± 1.08^e^	0.13 ± 0.01^d^	0.15 ± 0.06^f^	0.10 ± 0.01^d^
	Mu26	9.71 ± 0.28^d^	2.98 ± 0.12^cb^	4.64 ± 0.59^d^	1.74 ± 0.10^c^	11.11 ± 0.34^b^	0.29 ± 0.06^c^	0.44 ± 0.05^d^	0.21 ± 0.01^c^
	DM	8.07 ± 0.77^e^	3.19 ± 0.24^cb^	5.51 ± 0.60^d^	1.93 ± 0.18^c^	6.76 ± 0.69^d^	0.18 ± 0.03^d^	0.27 ± 0.07^e^	0.16 ± 0.01^c^
	OE17	25.70 ± 1.00^a^	7.54 ± 1.11^a^	19.60 ± 1.58^a^	5.72 ± 0.78^a^	18.75 ± 0.98^a^	0.62 ± 0.05^a^	1.03 ± 0.07^a^	0.46 ± 0.03^a^
	OE26	22.54 ± 1.48^b^	6.77 ± 0.81^a^	16.71 ± 1.45^b^	5.10 ± 0.62^a^	10.04 ± 0.64^cb^	0.55 ± 0.02^a^	0.74 ± 0.03^b^	0.42 ± 0.33^a^

*For each level of NaCl concentration, the significant differences between means were shown with different letters at *P* < 0.05.*

#### Photosynthesis Capacity of Overexpress and Mutant Plants

##### Carbohydrates content

Total carbohydrates content also decreased in all genotypes studied with increasing NaCl concentration (Table 2). In all concentrations of 50, 100, and 150 mM NaCl, OE17 and Mu17 plants showed a significant increase and decrease in the content of total carbohydrate content as compared to Col-0, respectively (Table 2). Moreover, simultaneous knockout mutation of *PAP17* and *PAP26* gens resulted in significantly lower accumulation of total carbohydrates in DM genotypes compared to that of WT plants in the three concentrations (Table 2). Interestingly, such results were not observed in OE26 and Mu26 genotypes (Table 2).

**FIGURE 1:**
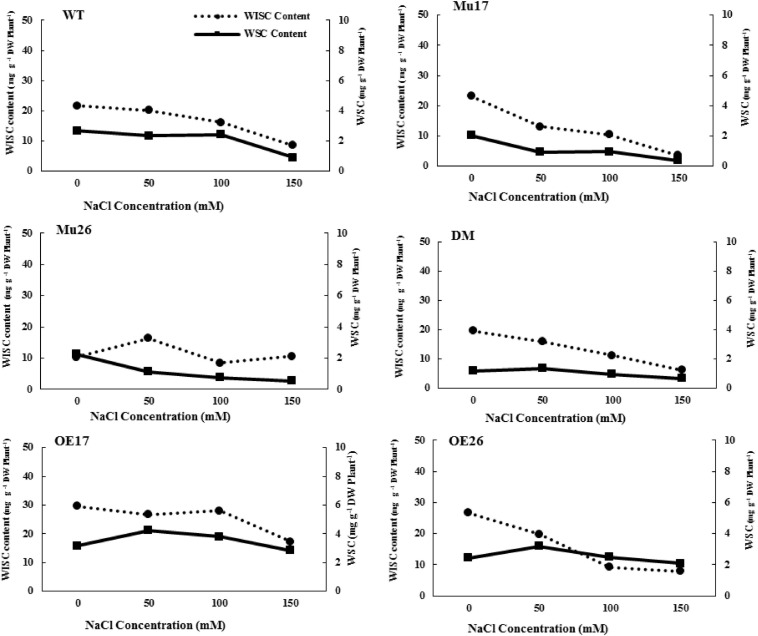
Trend of water-soluble carbohydrate (WSC) and water-insoluble carbohydrate (WISC) on genotypes subjected to NaCl stress. Wild type (WT), overexpress (OE), single mutant (Mu) and double mutant (DM) genotypes of *Arabidopsis thaliana* purple acid phosphatase 17 (*AtPAP17*) and 26 (*AtPAP26*) genes.

Further investigations showed that water-soluble and insoluble carbohydrate content also decreased in WT plants with increasing NaCl concentration (Figure 1). Despite this, the rate of reduction of soluble carbohydrate content was significantly lower (*t* < 0.05) than that for insoluble carbohydrate at the same conditions (Figure 1). Increasing NaCl concentration up to 50 mM, nonetheless, the rate of soluble carbohydrate increased in the overexpressed genotypes (b_*OE17*_ = 0.75 and b_*OE26*_ = 0.52). In fact, the trend of soluble carbohydrate in OE plants was significantly (*t* < 0.05) in the opposite direction to that in WT seedlings (b_*WT*_ = −0.73) (Figure 1). Rising NaCl concentration to 100 and 150 mM led to a decrease in soluble carbohydrates of OE genotypes; however, the reduction was significantly lower (b_*OE17*_ = −0.14 and b_*OE26*_ = 0.20, *t* < 0.05) than that in WT plants (b_*WT*_ = 0.82) (Figure 1).

The soluble carbohydrate content was significantly 2.16-fold and 1.33-fold higher in OE17 and OE26 than that in WT plants under 150 mM NaCl, respectively (Figure 1). Although overexpression of PAP26 has not resulted in significant changes in the total carbohydrate amount, the soluble carbohydrate content of OE26 plants was significantly higher than that in WT seedlings under 150 mM NaCl (Figure 1). The knockout mutation of *PAP17* and *PAP26* caused a significant decrease in soluble carbohydrates of Mu17, Mu26, and DM plants as compared to Col-0 in 150 mM NaCl (Figure 1).

##### Photosynthetic pigments stability

In all genotypes, chlorophyll *a* content decreased with increasing NaCl concentration in the culture media (Table 2). With rising NaCl concentration from 0 to 150 mM, the overexpressed and mutant genotypes showed the lowest and highest reduction in chlorophyll *a* content compared to that in WT plants (Table 2). Increasing of NaCl level from 0 to 150 mM led to a decrease in chlorophyll *b* and carotenoids content in all genotypes studied. However, the OE and mutant genotypes exhibited the lowest and highest reduction in those compared with WT plants (Table 2). According to the results, OE17 and OE26 seedlings showed significantly higher chlorophyll *a*, *b*, and carotenoid content in 150 mM NaCl, while the relative contents were significantly lower in mutant genotypes than those in wild-type plants (Table 2).

### The Cellular Role of AtPAP17 and AtPAP26 Under Salt Stress

#### Facilitating Water Retention

Overproduction of compatible organic solutes is one of the most common plants’ responses to physiological water deficit that are known to accumulate under salt stress in many crops (Munns and Tester, 2008). Here, with increasing NaCl concentration in the culture media, proline (shoots and roots) and glycine betaine content increased in all genotypes gradually, but differently for each genotype (Table 3). The overexpression of *AtPAP17* and *AtPAP26* genes led to a significant increase in proline (shoots and roots) and glycine betaine content in OE17 and OE26 plants. The relative contents, however, were significantly lower in Mu17, Mu26, and DM plants as compared to those in WT seedlings at 150 mM NaCl concentration (Table 3). Interestingly, the proline content was significantly more concentrated (*t* < 0.01) in roots than in shoots at 150 mM NaCl (Table 3).

**TABLE 3:** Means (±SE) of the physiological and biochemical parameters in genotypes subjected to the NaCl concentrations.

	Genotype	Proline content (μmol g^–1^ FW plant^−1^		GB content (μmol g^–1^ FW plant^–1^)	MDA content (μmol mg^–1^ FW	H_2_O_2_ content (μmol mg^–1^ FW)	APX (U g^–1^ FW)	POX (U g^–1^ FW)	CAT (U g^–1^ FW)
		Shoot	Root						
0 mM	WT	3.05 ± 0.21^b^	5.08 ± 0.47^c^	1.97 ± 0.52^a^	1.29 ± 0.10^cb^	0.08 ± 0.01^ab^	0.00 ± 0.00^c^	3.02 ± 0.09^d^	1.15 ± 0.25^e^
NaCl	Mu17	3.01 ± 0.42^b^	5.02 ± 0.52^c^	1.94 ± 0.38^a^	1.40 ± 0.05^b^	0.09 ± 0.01^ab^	1.25 ± 0.32^a^	6.24 ± 0.47^b^	4.60 ± 0.23^c^
	Mu26	2.09 ± 0.16^c^	3.48 ± 0.26^d^	1.81 ± 0.13^a^	1.65 ± 0.15^a^	0.08 ± 0.00^b^	0.85 ± 0.54^ab^	5.18 ± 0.26^c^	5.23 ± 0.32^b^
	DM	2.31 ± 0.20^c^	4.72 ± 0.64^c^	2.00 ± 0.1^7a^	1.34 ± 0.10^cb^	0.10 ± 0.03^ab^	0.40 ± 0.16^bc^	7.18 ± 0.11^a^	6.50 ± 0.57^a^
	OE17	1.39 ± 0.30^d^	8.10 ± 0.75^a^	1.20 ± 0.47^a^	1.19 ± 0.10^c^	0.08 ± 0.01^b^	0.00 ± 0.00^c^	2.94 ± 0.12^d^	1.89 ± 0.27^d^
	OE26	4.17 ± 0.52^a^	6.95 ± 0.67^b^	2.27 ± 0.15^a^	1.34 ± 0.26^cb^	0.10 ± 0.01^a^	0.00 ± 0.00^c^	2.74 ± 0.33^d^	1.91 ± 0.07^d^
50 mM	WT	17.45 ± 1.07^c^	23.58 ± 1.59^c^	8.10 ± 0.34^b^	4.75 ± 0.49^b^	0.12 ± 0.22^ab^	1.02 ± 0.22^b^	5.82 ± 0.91^e^	6.67 ± 0.66^d^
NaCl	Mu17	7.92 ± 0.53^e^	13.20 ± 1.28^e^	4.28 ± 0.56^c^	4.44 ± 0.41^cb^	0.11 ± 0.01^ab^	1.95 ± 0.24^a^	15.76 ± 0.26^a^	7.49 ± 0.29^c^
	Mu26	9.20 ± 0.33^ed^	15.33 ± 1.55^e^	4.97 ± 1.07^c^	5.94 ± 1.07^a^	0.12 ± 0.23^ab^	1.88 ± 0.44^a^	14.24 ± 0.54^b^	8.64 ± 0.32^b^
	DM	10.34 ± 1.18^d^	17.24 ± 2.24^d^	5.59 ± 0.49^c^	4.24 ± 0.18^cb^	0.12 ± 0.19^ab^	2.18 ± 0.50^a^	12.51 ± 0.64^c^	11.66 ± 0.91^a^
	OE17	26.41 ± 0.39^a^	35.33 ± 0.91^a^	14.27 ± 1.14^a^	3.00 ± 0.31^cd^	0.11 ± 0.01^b^	0.71 ± 0.10^b^	5.23 ± 0.39^e^	2.80 ± 0.56^f^
	OE26	23.95 ± 1.05^b^	30.65 ± 1.25^b^	12.94 ± 1.19^a^	3.57 ± 0.17^d^	0.13 ± 0.01^a^	0.90 ± 0.43^b^	7.55 ± 0.63^d^	5.24 ± 0.31^e^
100 mM	WT	17.77 ± 0.60^c^	30.48 ± 0.64^c^	9.60 ± 0.32^b^	5.94 ± 0.05^d^	0.17 ± 0.01^b^	1.97 ± 0.80^cb^	10.77 ± 0.47^d^	8.09 ± 0.69^c^
NaCl	Mu17	12.64 ± 0.80^e^	21.06 ± 0.96^ed^	6.83 ± 1.05^c^	8.11 ± 0.49^c^	0.16 ± 0.02^b^	1.65 ± 0.16^c^	11.86 ± 1.18^d^	9.27 ± 0.33^b^
	Mu26	11.40 ± 0.30^f^	18.99 ± 0.50^e^	6.16 ± 0.16^c^	10.54 ± 0.54^a^	0.21 ± 0.03^a^	2.13 ± 0.40^b^	7.97 ± 0.24^e^	5.04 ± 0.43^d^
	DM	14.27 ± 0.63^d^	23.79 ± 0.90^d^	7.72 ± 1.45^c^	9.71 ± 0.67^b^	0.25 ± 0.04^a^	1.96 ± 0.19^cb^	15.26 ± 0.61^c^	9.35 ± 0.63^b^
	OE17	30.45 ± 0.73^b^	44.96 ± 1.93^b^	16.46 ± 1.47^a^	4.39 ± 0.42^e^	0.16 ± 0.02^b^	2.99 ± 0.39^a^	21.20 ± 0.55^a^	11.34 ± 0.58^a^
	OE26	32.94 ± 0.92^a^	51.42 ± 2.89^a^	17.80 ± 0.49^a^	3.82 ± 0.33^e^	0.13 ± 0.02^b^	2.68 ± 0.12^a^	17.66 ± 0.33^b^	10.56 ± 0.30^a^
150 mM	WT	23.67 ± 0.99^c^	45.24 ± 1.64^c^	12.80 ± 1.16^b^	7.96 ± 0.44^d^	0.26 ± 0.02^b^	1.82 ± 0.80^cb^	12.57 ± 0.65^c^	5.78 ± 0.52^c^
NaCl	Mu17	10.13 ± 0.71^e^	26.44 ± 2.12^f^	5.48 ± 0.38^d^	9.25 ± 0.67^c^	0.28 ± 0.03^b^	1.18 ± 0.32^cd^	5.98 ± 0.25^e^	2.95 ± 0.34^e^
	Mu26	11.90 ± 0.28^e^	31.42 ± 2.03^e^	6.43 ± 0.15^d^	13.28 ± 0.60^a^	0.31 ± 0.30^a^	0.83 ± 0.14^d^	6.24 ± 0.15^e^	3.75 ± 0.80^d^
	DM	18.28 ± 0.59^d^	36.26 ± 0.96^d^	9.88 ± 0.32^c^	11.52 ± 0.59^b^	0.32 ± 0.30^a^	1.51 ± 0.67^c^	9.60 ± 0.45^d^	1.31 ± 0.67^f^
	OE17	36.81 ± 2.03^a^	74.37 ± 3.55^a^	16.90 ± 1.11^a^	5.42 ± 0.80^e^	0.14 ± 0.30^c^	3.63 ± 0.38^a^	23.24 ± 0.56^a^	13.27 ± 0.58^a^
	OE26	29.12 ± 2.63^b^	60.11 ± 2.94^b^	15.74 ± 1.31^a^	6.20 ± 0.09^e^	0.14 ± 0.01^c^	2.42 ± 0.46^b^	16.05 ± 1.46^b^	9.69 ± 0.92^b^

*For each level of NaCl concentration, the significant differences between means were shown with different letters at *P* < 0.05.*

#### Maintenance of Cellular Stability

##### Lipid peroxidation assay

In all genotypes, increasing NaCl concentration (50, 100, and 150 mM) resulted in a gradual increase of MDA, as a biomarker of lipid peroxidation (Table 3). This alteration could represent the increase of salt stress sensing and/or low efficiency in ROS scavenging and cell damage management. Examination of the responses to salt stress displayed dissimilar feedback in the genotypes where the overexpressed and mutant plants showed dramatically the lowest and the highest increase in MDA amount compared to WT plants, with increasing NaCl concentration (Table 3). In 100 and 150 mM NaCl, the MDA content of OE17 and OE26 plants were also significantly lower than to Col-0, while Mu17, Mu26, and DM seedlings had significantly more accumulation of MDA content compared to the control ones (Table 3).

##### Hydrogen peroxide-induced response

Salt stress (50, 100, and 150 mM NaCl) also increased H_2_O_2_ accumulation in all genotypes. The lowest increase belonged to OE26 and OE17 plants, but Mu26 showed the most increase in H_2_O_2_ accumulation among other genotypes (Table 3). Also, the overexpressed plants (OE17 and OE26) showed a significantly lower amount of H_2_O_2_, while Mu26 and DM plants had more amount of H_2_O_2_ compared to WT seedlings at 150 mM NaCl (Table 3).

##### Oxidative stress adaptation

###### Enzymatic antioxidants activity

With rising NaCl concentration from 0 to 150 mM, the highest increase in APX, CAT, and POX activities belonged to the overexpressed plants, while the mutant genotypes showed the lowest increase compared to the wild-types (Table 3). In 150 mM NaCl, overexpression and knockout mutation of *PAP17* and *PAP26* also resulted in significantly higher and lower activities in CAT and POX enzymes in overexpressed and mutant genotypes compared to WT seedlings, respectively (Table 3). Interestingly, the observed differences were more in OE17 shoots, and following that, OE26 shoots at the same condition (Table 3).

###### Non-enzymatic antioxidants activity

No significant differences were detected among genotypes in anthocyanin as well as flavonoid content in different conditions (data not shown).

#### Regulation of Na^+^ and K^+^ Fluxes

**FIGURE 2:**
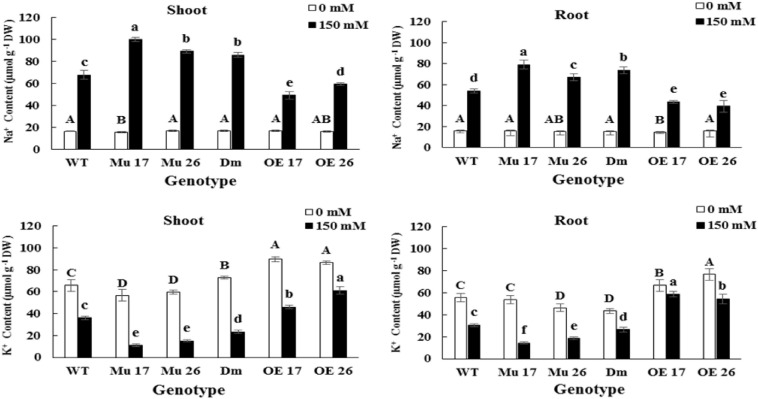
The Na^+^ and K^+^ accumulation in shoots and roots of genotypes subjected to 150 mM NaCl stress compared to no NaCl stress. Wild-type (WT), overexpress (OE), single mutant (Mu), and double mutant (DM) genotypes of *Arabidopsis thaliana* purple acid phosphatase 17 (*AtPAP17*) and 26 (*AtPAP26*) genes. The significant mean differences (*P* < 0.05) were separately shown at 0 and 150 mM NaCl concentrations with different uppercase and lowercase letters, respectively.

Rising NaCl concentration from 0 to 150 mM increased Na^+^ and decreased K^+^ content in both shoots and roots. The changes were different in the various genotypes where overexpressed plants showed the lowest increase and decrease in Na^+^ and K^+^ accumulation, respectively (Figure 2). On the other hand, the highest increase in Na^+^ accumulation, and a decrease in K^+^ accumulation belonged to the mutant plants compared to WT ones (Figure 2).

The accumulation of Na^+^ in shoots was on average 20.01% (*t* < 0.01) more than that in roots, at 150 mM NaCl. In this concentration of NaCl, overexpression of *PAP17* and *PAP26* also caused a significantly lower accumulation of Na^+^ in shoots and roots of OE genotypes compared to that in WT plants (Figure 2). The knockout mutation of these two genes resulted in a significant accumulation of Na^+^ in shoots and roots of all mutant plants compared with WTs at the same condition (Figure 2). Despite these results, the rate of accumulation of K^+^ was more in shoots and roots of OE and less in mutant plants compared to WT seedlings following 150 mM NaCl (Figure 2).

**FIGURE 3:**
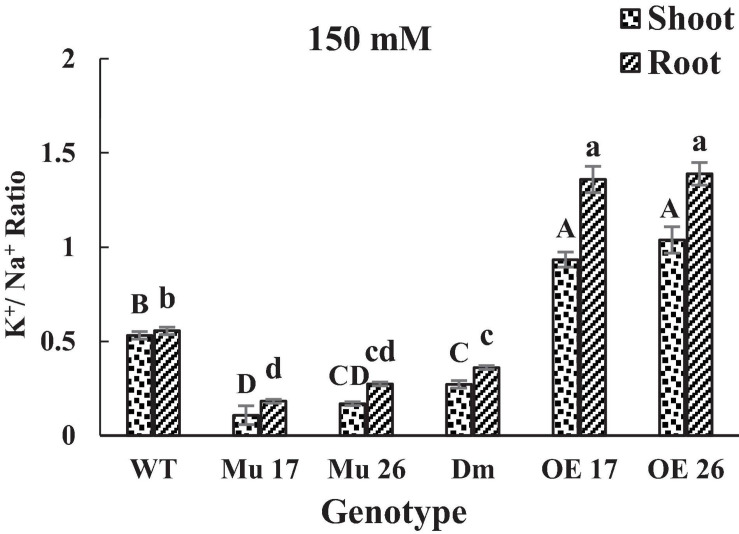
The K^+^/ Na^+^ ratio in shoots and roots of genotypes at 150 mM NaCl stress. Wild-type (WT), overexpress (OE), single mutant (Mu), and double mutant (DM) genotypes of *Arabidopsis thaliana* purple acid phosphatase 17 (*AtPAP17*) and 26 (*AtPAP26*) genes. The significant mean differences (*P* < 0.05) were separately shown for shoot and root with different uppercase and lowercase letters, respectively.

Finally, the consequence of PAP17 and PAP26 activities in the regulation of Na^+^ and K^+^ accumulation modified K^+^/Na^+^ ratio both in shoots and roots (Figure 3) where the K^+^/Na^+^ ratio of overexpressed plants was significantly higher than WT plants under 150 mM NaCl. Conversely, the relative ratio in shoots and roots of Mu17, Mu26, and DM plants was significantly less than the control ones at the same NaCl concentration (Figure 3). The correlation coefficient between K^+^/Na^+^ ratio and dry seedling weight was estimated 0.94 (*P* < 0.01), while 0.82 (*P* < 0.01) correlation was detected between Na^+^ content and seedling dry weight.

### Transcriptional Profiling of Important Genes Involved in Plants Na^+^ Management

**FIGURE 4:**
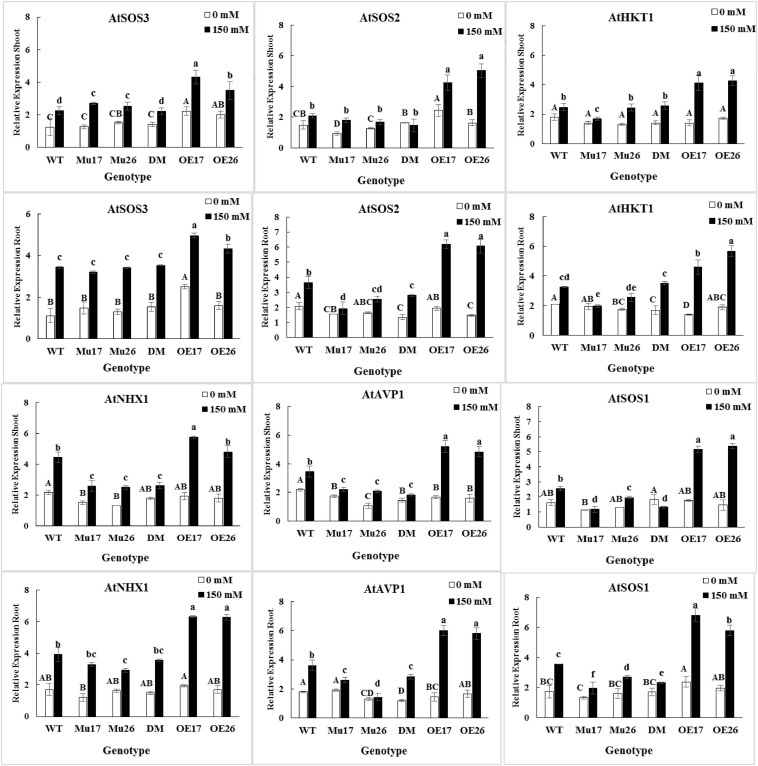
The expression level of mentioned genes in shoots and roots of genotypes subjected to 150 mM NaCl stress compared to no NaCl stress. Wild-type (WT), overexpress (OE), single mutant (Mu), and double mutant (DM) genotypes of *Arabidopsis thaliana* purple acid phosphatase 17 (*AtPAP17*) and 26 (*AtPAP26*) genes. The significant mean differences (*P* < 0.05) were separately shown at 0 and 150 mM NaCl concentrations with different uppercase and lowercase letters, respectively.

The expression level of *SOS3*, *SOS2*, *HKT1*, *NHX1*, *AVP1*, and *SOS1* genes were up-regulated by salt stress (0–150 mM NaCl) in the roots and shoots of all genotypes (Figure 4). The changes of the expression levels were higher in the roots (*t* < 0.01) compared to shoots at the concentration of NaCl. Thanks to the different genetic potential of genotypes, the increase in expression levels of the genes were proportionally dissimilar for each genotype, to respond to salinity stress.

In 150 mM NaCl, the expression level of *SOS3* gene was significantly higher in shoots and roots of the overexpressed plants as compared to the wild-type ones (Figure 4). The *SOS2* gene expression level was significantly higher in shoots and roots of OE plants than WT, while the roots of mutant plants showed the lowest relative expression value under 150 mM NaCl condition (Figure 4). The expression level of *HKT1* gene was significantly higher in both roots and shoots of OE17 and OE26 plants compared to WT plants at 150 mM NaCl. The significantly lower *HKT1* expression was detected in shoots and roots of Mu17 under the same sodium concentration (Figure 4). Among all seedlings growing under 150 mM NaCl, the OE17 (in shoots and roots) and OE26 (in shoots) exhibited significantly higher expression levels of *NHX1* gene compared to WT plants. However, the lowest expression level of the gene was significantly measured in shoots of all mutant genotypes (Mu17, Mu26, and DM), and roots of Mu26 genotype (Figure 4). The overexpression of PAP17 and PAP26 also significantly caused a higher expression level in *AVP1* gene in shoots and roots of the overexpressed plants compared to Col-0 in 150 mM NaCl. On the other hand, Knockout mutation of the genes in double-mutant plants resulted in significantly lower expression of *AVP1* gene in the plant’s shoot and root as compared to WT plants (Figure 4). Overexpression and knockout mutation of PAP17 and PAP26 also resulted significantly in more expression level of *SOS1* gene in both shoots and roots of OE plants and lower in mutant plants when compared to the control plants in 150 mM sodium concentration (Figure 4).

### Regulation of Phosphate Homeostasis

#### Total, Inorganic, and Organic Phosphate Content

To investigate systematically the role of PAP17 and PAP26 in phosphorus supply, total phosphate content, inorganic (Pi), and organic phosphate (Po) content were estimated in both shoots and roots of the plants treated with 0, 50, 100, and 150 mM NaCl. The total P content was obviously affected by salt stress in shoots and roots of genotypes studied while rising NaCl concentration to 50, 100, and 150 mM (Table 4). Our results clearly demonstrated that overexpression of *PAP17* and *PAP26* genes led to the lowest rate of reduction of total P content in OE seedling under saline conditions (Table 4). At the same condition, however, the knockout mutation of the genes caused the most rate of total P content reduced in mutant plants (Table 4). Furthermore, the total P content in wild-type plants exposed to 150 mM NaCl was approximately 27% lower (*t* < 0.01) in shoots than roots. The overexpression of *PAP17* and *PAP26* created significantly higher total P content in OE plants compared to WT plants at 150 mM NaCl. In contrast, the knockout mutation of the genes resulted in significantly lower total P content in shoots and roots of Mu17, Mu26, and DM genotypes compared to WT plants at the same concentration (Table 4).

**TABLE 4:** Means (±SE) of the total P, free Pi, and P organic contents in genotypes subjected to the NaCl concentrations.

	Genotype	Total P content (μmol g^–1^ DW plant^–1^)		Pi content (μmol g^–1^ DW plant^–1^)		Po content (μmol g^–1^ DW plant^–1^)		APase activity (U mg^–1^ protein)	
		Shoot	Root	Shoot	Root	Shoot	Root	Shoot	Root
0 mM	WT	28.83 ± 0.79^b^	36.56 ± 1.89^c^	6.49 ± 0.36^cd^	6.71 ± 0.66^c^	22.34 ± 0.50^c^	29.84 ± 2.31^c^	0.54 ± 0.05^c^	1.02 ± 0.15^ab^
NaCl	Mu17	20.87 ± 0.49^c^	27.68 ± 2.11^d^	5.59 ± 0.38*d*^e^	6.46 ± 0.64^c^	15.28 ± 0.46^d^	21.22 ± 1.91^d^	0.51 ± 0.03^c^	0.64 ± 0.16^b^
	Mu26	25.19 ± 2.36^cb^	24.96 ± 0.71^e^	5.89 ± 0.32^e^	4.27 ± 0.25^d^	19.31 ± 2.12^e^	20.69 ± 0.47^d^	0.59 ± 0.02^c^	0.71 ± 0.02^b^
	DM	27.34 ± 0.34^b^	22.79 ± 1.16^e^	7.19 ± 0.22^c^	3.90 ± 0.17^d^	20.15 ± 0.55^cd^	18.89 ± 0.99^d^	0.76 ± 0.08^b^	0.55 ± 0.15^ab^
	OE17	42.42 ± 3.84^a^	50.31 ± 1.19^a^	9.52 ± 0.78^a^	8.04 ± 0.26^b^	32.90 ± 1.95^a^	42.28 ± 1.07^a^	1.55 ± 0.01^a^	1.70 ± 0.36^a^
	OE26	37.90 ± 1.40^a^	42.67 ± 0.89^b^	8.54 ± 0.73^b^	9.23 ± 0.21^a^	29.36 ± 1.01^b^	33.44 ± 0.69^b^	1.57 ± 0.05^a^	1.31 ± 0.12^a^
50 mM	WT	19.76 ± 0.68^c^	22.61 ± 0.52^d^	3.92 ± 1.00^c^	4.58 ± 0.78^cd^	15.84 ± 0.71^c^	18.03 ± 0.91^d^	0.87 ± 0.11^b^	1.20 ± 0.01^c^
NaCl	Mu17	37.73 ± 0.12^a^	33.55 ± 1.93^b^	9.73 ± 0.70^b^	5.87 ± 0.12^cb^	28.00 ± 0.81^a^	27.68 ± 1.94^b^	0.49 ± 0.07^d^	1.23 ± 0.12^c^
	Mu26	14.39 ± 1.92^d^	29.41 ± 1.94^c^	4.74 ± 0.64^c^	5.53 ± 0.16^cb^	9.65 ± 0.44^e^	23.88 ± 1.87^c^	0.67 ± 0.03^c^	0.90 ± 0.14^d^
	DM	17.21 ± 1.27^cd^	20.07 ± 1.06^e^	4.65 ± 0.43^c^	3.69 ± 0.50^d^	12.56 ± 0.89^d^	16.38 ± 1.42^d^	0.97 ± 0.12^b^	1.32 ± 0.11^c^
	OE17	39.26 ± 3.37^a^	52.92 ± 2.01^a^	11.61 ± 1.06^a^	7.02 ± 0.91^b^	27.64 ± 1.57^a^	45.90 ± 3.05^a^	2.19 ± 0.0^6a^	2.27 ± 0.09^a^
	OE26	30.94 ± 1.52^b^	33.65 ± 0.76^b^	9.71 ± 0.69^b^	10.36 ± 1.23^a^	21.22 ± 1.97^b^	22.69 ± 2.15^c^	2.30 ± 0.1^2a^	1.85 ± 0.08^b^
100 mM	WT	15.50 ± 0.61^c^	18.68 ± 2.35^c^	3.68 ± 0.40^b^	3.48 ± 0.69^b^	11.82 ± 0.95^c^	15.20 ± 1.80^c^	1.43 ± 0.07^c^	1.56 ± 0.11^c^
NaCl	Mu17	5.49 ± 0.37^e^	15.01 ± 3.77^c^	2.46 ± 0.35^c^	3.70 ± 0.30^b^	3.03 ± 0.08^e^	11.31 ± 1.43^d^	0.53 ± 0.13^e^	0.80 ± 0.05^d^
	Mu26	11.34 ± 0.82^d^	17.76 ± 0.15^c^	3.23 ± 0.20^cb^	3.29 ± 0.29^b^	8.11 ± 0.62^d^	14.47 ± 0.39^c^	0.82 ± 0.2^d^	0.57 ± 0.11^d^
	DM	11.79 ± 0.85^d^	18.83 ± 1.54^c^	3.21 ± 0.27^cb^	2.75 ± 0.59^b^	8.58 ± 0.63^d^	16.08 ± 1.04^c^	1.08 ± 0.22^d^	0.81 ± 0.15^d^
	OE17	36.25 ± 0.79^a^	48.17 ± 1.85^a^	7.18 ± 0.53^a^	6.77 ± 0.73^a^	29.07 ± 0.42^a^	41.40 ± 1.30^a^	2.66 ± 0.23^a^	2.55 ± 0.16^b^
	OE26	27.79 ± 2.01^b^	29.36 ± 1.35^b^	7.87 ± 0.64^a^	6.78 ± 0.79^a^	19.92 ± 1.41^b^	22.10 ± 2.31^b^	2.38 ± 0.08^b^	3.13 ± 0.30^a^
150 mM	WT	9.02 ± 1.09^c^	12.41 ± 1.37^c^	3.23 ± 0.11^c^	2.65 ± 0.56^b^	5.79 ± 1.18^c^	9.76 ± 0.83^c^	2.07 ± 0.04^b^	2.06 ± 0.16^c^
NaCl	Mu17	4.84 ± 0.51^ed^	8.94 ± 0.36^d^	0.73 ± 0.23^f^	0.91 ± 0.22^c^	4.10 ± 0.28^cd^	8.03 ± 0.25^c^	0.85 ± 0.06^c^	1.13 ± 0.03^d^
	Mu26	3.84 ± 0.19^e^	4.76 ± 0.44^e^	1.35 ± 0.11^e^	0.98 ± 0.07^c^	2.48 ± 0.22^d^	3.78 ± 0.50^e^	0.50 ± 0.10^d^	0.59 ± 0.01^e^
	DM	5.82 ± 0.43^d^	8.43 ± 0.70^d^	2.09 ± 0.19^d^	1.71 ± 0.21^c^	3.73 ± 0.23^d^	6.72 ± 0.59^d^	0.78 ± 0.14^c^	0.78 ± 0.04^e^
	OE17	22.18 ± 1.32^a^	31.68 ± 1.76^a^	4.94 ± 0.29^a^	6.11 ± 0.16^a^	17.24 ± 1.61^a^	25.57 ± 1.92^a^	2.80 ± 0.12^a^	3.67 ± 0.11^a^
	OE26	18.27 ± 1.12^b^	26.12 ± 2.18^b^	6.56 ± 0.47^b^	5.42 ± 0.65^a^	11.71 ± 0.66^b^	19.75 ± 2.66^b^	2.15 ± 0.15^b^	3.04 ± 0.20^b^

*For each level of NaCl concentration, the significant differences between means were shown with different letters at *P* < 0.05.*

The evaluation of total P components (Pi and Po content) clearly showed that increasing NaCl concentrations also led to a gradual decrease in relative contents of P components in WT plants, as control plants (Table 4). The overexpressed and Mu26 genotypes showed the lowest and highest reduction in Pi and Po content in both shoots and roots compared to the wild-types, respectively (Table 4). The overexpression and knockout mutation of *PAP17* and *PAP26* also caused a significant increase and decrease of Pi and Po content in shoots and roots of OE and mutant genotypes when compared to WT seedlings in 150 mM NaCl, respectively (Table 4).

The correlation coefficient of Pi and total carbohydrate was 0.66 (*P* < 0.01) in 150 mM NaCl. Still, the correlation between Pi and soluble carbohydrate in the same concentration of salt was observed to be 0.82 (*P* < 0.01).

#### Phosphatase Activity

The APase activity of wild-type plants increased 0.62-fold, 1.6-fold, and 2.8-fold in the shoots as well as 0.17-fold, 0.52-fold, and 1.01-fold in the roots under 50, 100, and 150 mM NaCl, respectively, compared to the normal condition (0 mM) (Table 4). Moreover, the increase in the APase activity of WT plants was detected to be significantly higher in the shoots (*t* < 0.01) than the roots at 150 mM NaCl. In 150 mM NaCl, OE17 and OE26 plants showed significantly higher APase activity (shoots and roots) compared to WT plants (Table 4). The reverse results were observed for shoots and roots of Mu17, Mu26, and DM seedlings at this concentration of NaCl (Table 4).

### NaCl Stress Up-Regulates the Expression of AtPAP17 and AtPAP26 Genes

**FIGURE 5:**
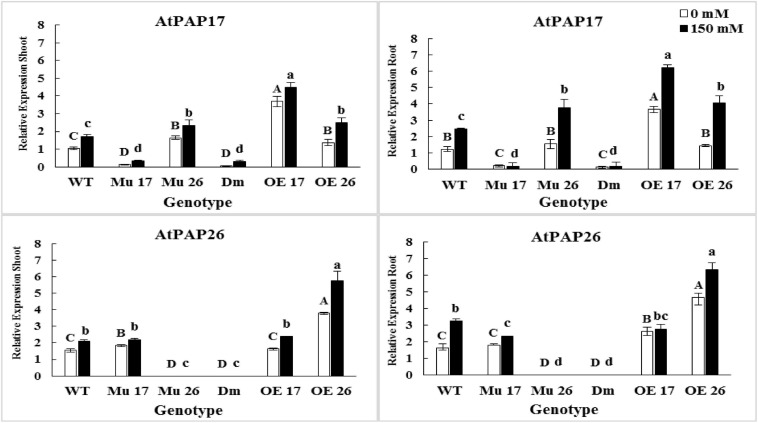
The expression level of *AtPAP17* and *AtPAP26* genes in shoots and roots of genotypes subjected to 150 mM NaCl stress compared to no NaCl stress. Wild-type (WT), overexpress (OE), single mutant (Mu), and double mutant (DM) genotypes of *Arabidopsis thaliana* purple acid phosphatase 17 (*AtPAP17*) and 26 (*AtPAP26*) genes. The significant mean differences (*P* < 0.05) were separately shown at 0 and 150 mM NaCl concentrations with different uppercase and lowercase letters, respectively.

Through severe salinity stress (150 mM NaCl), the expression level of *PAP17* and *PAP26* genes were strongly induced in both roots and shoots of WT plants (Figure 5). Indeed, rising NaCl from 0 to 150 mM led to an increase in the PAP17 and PAP26 expression levels by 66 and 37% in shoots as well as 99 and 100% in roots of WT plants (Figure 5). When overexpressed genotypes subjected to 150 mM NaCl, the expression level of *PAP17* were detected to be 21 and 69% more in the shoots and roots of OE17 plants, respectively, compared to those under normal condition (Figure 5). At the same concentration of salt, the expression level of *PAP26* was 51% higher in shoots and 35% higher in roots of OE26 plants compared to the plants in normal condition. Besides, the expression level of *PAP17* and *PAP26* were detected to be significantly higher in both shoots and roots of OE17 and OE26 compared to wild-type plants at 150 mM NaCl, respectively (Figure 5).

**FIGURE 6:**
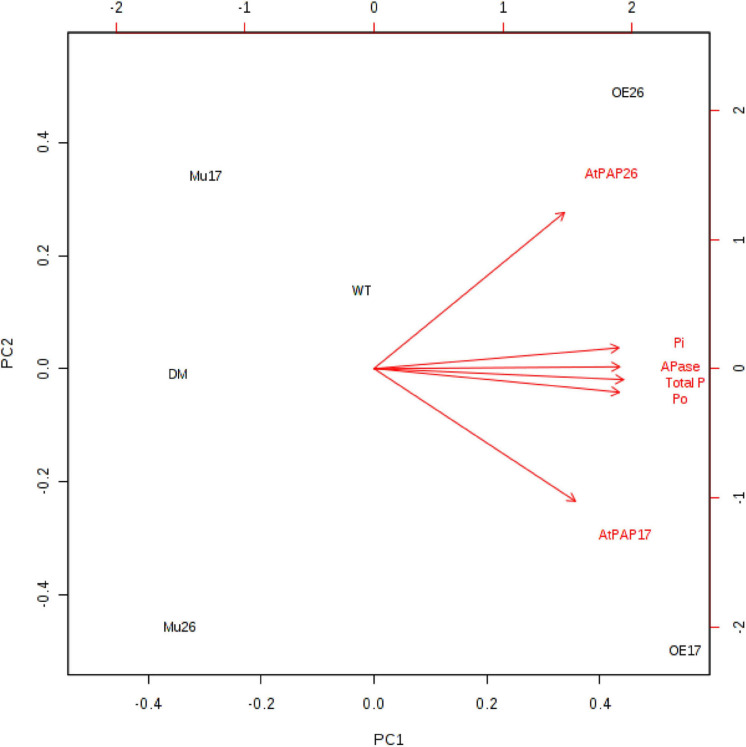
Biplot analysis of the expression level of *AtPAP17* and *AtPAP26* genes, APase activity, total phosphate, inorganic (Pi), and organic phosphate (Po) content in the examined genotypes to show the relationship at 150 mM NaCl stress. Wild type (WT), overexpress (OE), single mutant (Mu), and double mutant (DM) genotypes of *Arabidopsis thaliana* purple acid phosphatase 17 (*AtPAP17*) and 26 (*AtPAP26*) genes.

Further investigation showed a significant (*P* < 0.01) correlation between the expression levels of *PAP17* and *PAP26* genes and APase activity. The APase activity was also correlated with total, inorganic, and organic phosphate content in 150 mM NaCl (Figure 6).

### AtPAP17 and AtPAP26 Improve Seed Yield

To investigate the effect of AtPAP17 and AtPAP26 on seed yield, genotypes were treated with different levels of NaCl under *in vivo* condition. Our results showed that the flowering percent in double-mutant plants was significantly lower than that in wild-type plants at 150 mM NaCl (Table 5). At the beginning of the stress period, the flowering percent and pod number in DM genotype were not significantly different compared to WT seedlings at 100 mM NaCl. No seeds, nevertheless, were observed in double-mutant plants at the seedling stage in pods at this condition (with more salt stress duration) (Table 5). In addition, no seeds were produced in pods of Mu26 and DM genotypes under longer salt stress duration at the highest sodium concentration (150 mM NaCl). The seed number per pod also was significantly lower in Mu17 and higher OE17 genotypes compared to WT plants under 150 mM NaCl condition (Table 5). Thus, no seed and 1000 seed weight were recorded in DM (at both 100 and 150 mM NaCl) and Mu26 (at 150 mM NaCl) genotypes as they could not produce any seed in high saline conditions (Table 5). However, Mu17 plants produced seeds, which had the characteristics of non-living seeds under long-term saline (150 mM NaCl) condition. In spite of these results, OE17 and OE26 genotypes showed significantly higher in 1000 seed weight compared with WT plants at the same condition (Table 5). Hence, the total seed yield of Mu17 and DM plants was significantly lower than those in the control group under 50 mM NaCl. Although the total seed yield of Mu17 genotype was lower than that in WT plants, the relative amount in OE17 and OE26 genotypes were significantly more as compared to that in WT plant at 100 mM NaCl (Table 5). In addition, DM genotype did not obtain any seeds under 100 mM NaCl. The total seed yield of OE17 and OE26 genotypes were significantly higher than those in WT plants, whereas the seed yield was not maintained well in Mu17, Mu26, and DM genotypes at 150 mM NaCl (Table 5).

**TABLE 5:** Means (±SE) of the yield and yield components in genotypes subjected to the NaCl concentrations.

	Genotype	Flowering %	Number of pods plant^–1^	Number of seed pods^–1^	1000 seed weight (mg)	Total seed yield (mg plant^–1^)
0 mM	WT	65.04 ± 5.1^b^	6.44 ± 1.3^a^	36.00 ± 0.6^b^	17.00 ± 0.2^b^	3.92 ± 0.8^ab^
NaCl	Mu17	25.95 ± 1.0^c^	2.66 ± 0.2^b^	35.00 ± 1.7^b^	15.73 ± 0.5^c^	3.47 ± 0.1^b^
	Mu26	61.62 ± 3.6^b^	6.51 ± 0.3^a^	34.67 ± 1.2^b^	16.40 ± 0.1^b^	3.70 ± 0.2^ab^
	DM	71.11 ± 4.4^b^	8.78 ± 0.9^a^	36.33 ± 0.3^b^	15.20 ± 0.1^c^	4.86 ± 0.5^ab^
	OE17	94.44 ± 5.5^a^	8.42 ± 1.2^a^	40.67 ± 1.2^a^	18.07 ± 0.4^a^	5.27 ± 0.6^a^
	OE26	76.67 ± 6.7^b^	7.89 ± 0.4^a^	33.33 ± 0.7^b^	18.07 ± 0.2^a^	4.77 ± 0.3^ab^
50 mM	WT	51.19 ± 1.9^b^	6.70 ± 1.1^a^	32.00 ± 1.5^b^	15.67 ± 0.2^b^	3.32 ± 0.4^a^
NaCl	Mu17	74.60 ± 12.9^ab^	3.47 ± 0.8^b^	17.67 ± 1.4^c^	11.93 ± 0.8^c^	0.71 ± 0.1^b^
	Mu26	75.84 ± 7.3^b^	5.71 ± 0.6^ab^	28.00 ± 1.5^b^	13.00 ± 0.3^c^	2.10 ± 0.3^ab^
	DM	81.57 ± 4.2^b^	6.01 ± 0.8^ab^	21.00 ± 1.0^c^	9.00 ± 0.2^d^	1.12 ± 0.1^b^
	OE17	90.58 ± 1.2^b^	5.81 ± 1.1^ab^	36.67 ± 1.2^a^	17.13 ± 0.8^a^	3.69 ± 0.8^a^
	OE26	88.64 ± 7.3^b^	6.59 ± 0.8^a^	38.33 ± 0.7^a^	15.13 ± 0.5^b^	2.90 ± 0.8^a^
100 mM	WT	81.82 ± 2.0^ab^	4.27 ± 0.5^b^	28.33 ± 3.5^b^	12.06 ± 1.8^b^	1.45 ± 0.3^c^
NaCl	Mu17	71.14 ± 5.3^b^	4.35 ± 0.3^b^	22.33 ± 1.4^c^	9.733 ± 0.3^b^	0.94 ± 0.1^d^
	Mu26	71.15 ± 4.5^b^	6.75 ± 0.4^a^	25.67 ± 0.3*b*^c^	11.40 ± 0.4^b^	1.73 ± 0.0^bc^
	DM	70.85 ± 7.9^b^	5.01 ± 0.6^b^	0.00 ± 0.0^d^	0.00 ± 0.0^c^	0.00 ± 0.0^e^
	OE17	90.11 ± 5.0^a^	4.12 ± 0.2^b^	26.67 ± 0.3^bc^	15.26 ± 0.2^a^	2.14 ± 0.1^ab^
	OE26	69.80 ± 1.6^b^	5.33 ± 0.3^b^	34.33 ± 0.7^a^	12.00 ± 0.4^b^	2.19 ± 0.1^a^
150 mM	WT	54.44 ± 10.9^a^	3.23 ± 0.4^bc^	21.33 ± 2.1^b^	9.60 ± 0.3^c^	0.67 ± 0.1^c^
NaCl	Mu17	47.59 ± 5.5^ab^	3.05 ± 0.3^c^	10.00 ± 0.0^c^	0.00 ± 0.0^d^	0.00 ± 0.0^d^
	Mu26	60.32 ± 5.2^a^	4.50 ± 0.4^a^	0.00 ± 0.0^d^	0.00 ± 0.0^d^	0.00 ± 0.0^d^
	DM	33.12 ± 12.0^b^	2.52 ± 0.2^c^	0.00 ± 0.0^d^	0.00 ± 0.0^d^	0.00 ± 0.0^d^
	OE17	64.50 ± 4.5^a^	3.90 ± 0.2^ab^	31.67 ± 1.2^a^	14.33 ± 1.0^a^	1.88 ± 0.2^a^
	OE26	65.48 ± 3.0^a^	3.22 ± 0.2^bc^	22.67 ± 0.3^b^	11.53 ± 0.6^b^	1.27 ± 0.1^b^

*For each level of NaCl concentration, the significant differences between means were shown with different letters at *P* < 0.05.*

Taken together, Mu17 and Mu26 (at 150 mM NaCl), and DM (at 100 and 150 mM NaCl) did not maintain total seed yield. Despite these results, the OE17 and OE26 genotypes also produced the highest total seed yield under 100 and 150 mM NaCl as well (Table 5).

### Complementary Analysis

**FIGURE 7:**
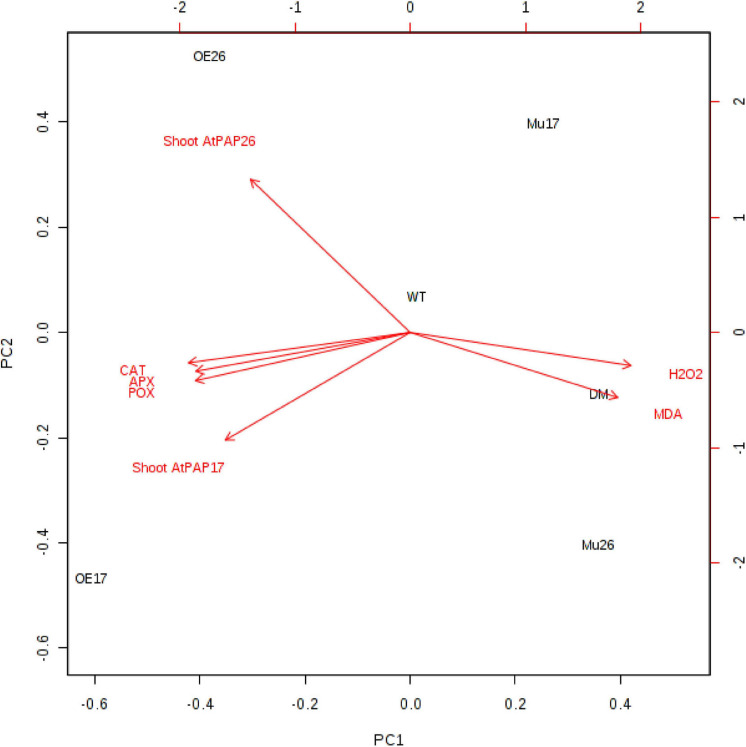
Biplot analysis of the expression level of *ATPAP17* and *AtPAP26* genes in shoot; catalase (CAT), guaiacol peroxidase (POX), and ascorbate peroxidase activities (APX); H_2_O_2_ and MAD accumulation in the examined genotypes to show the relationship at 150 mM NaCl stress. Wild type (WT), overexpress (OE), single mutant (Mu), and double mutant (DM) genotypes of *Arabidopsis thaliana* purple acid phosphatase 17 (*AtPAP17*) and 26 (*AtPAP26*) genes.

**FIGURE 8:**
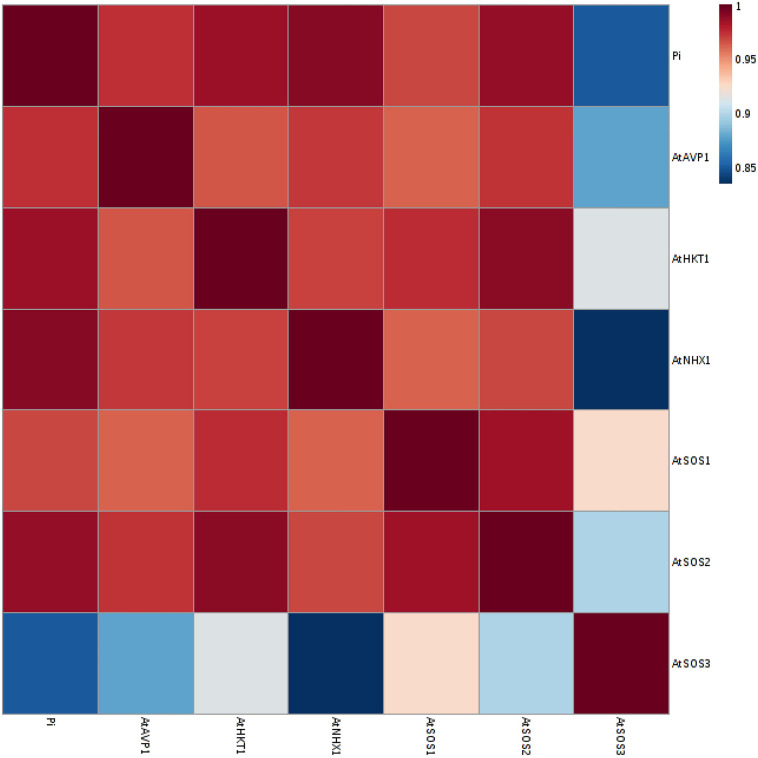
A Heat-map of correlations between the expression level of *AtSOS1*, *AtSOS2*, *AtSOS3*, *AtHKT1*, *AtVPV1*, and *AtNHX1* genes, and inorganic (Pi) content at 150 mM NaCl stress. The color key represents the quantile-normalized log10-transformed values. Dark red indicates high level whereas dark blue indicates low level.

**FIGURE 9:**
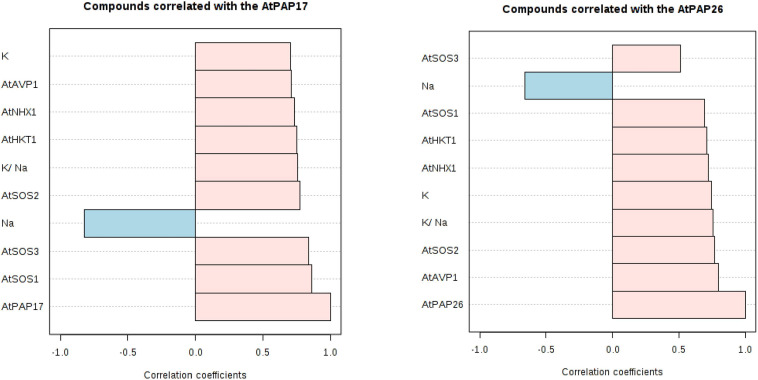
Correlation coefficients between the expression level of *AtPAP17* and *AtPAP26* genes and the expression level of *AtSOS1, AtSOS2, AtSOS3, AtHKT1, AtVPV1*, and *AtNHX1* genes; Na^+^ and K^+^ accumulation; K^+^/ Na^+^ ratio at 150 mM NaCl stress.

A principal component analysis (PCA) of Pearson’s correlation revealed that the expression level of *AtPAP17* and *AtPAP26* genes associated with the activity of APX, CAT, and POX enzymes involved in reducing H_2_O_2_ and MAD accumulation in 150 mM NaCl condition (Figure 7). In the present study, the correlation between the *PAP17* expression level and the activity of POX and CAT enzymes was observed to be 0.75 and 0.83 (*P* < 0.01), respectively. Furthermore, the correlation between *PAP26* expression level with POX and CAT enzyme activities was found to be 0.49 and 0.58 (*P* < 0.05), respectively, at 150 mM NaCl condition (Figure 7). A high and significant positive correlation was detected between the Pi content and the expression levels of *SOS3* (*r* = 0.69^∗∗^), *SOS2* (*r* = 0.89^∗∗^), *NHX1* (*r* = 0.91^∗∗^), *AVP1* (0.88^∗∗^), and *SOS1* (0.92^∗∗^) at 150 mM NaCl conditions (Figure 8). There were also significant correlations between the expression levels of *SOS3* and *SOS2* genes with *NHX1* (0.82 and 0.90, *P* < 0.01), *AVP1* (0.87 and 0.96, *P* < 0.01), and *SOS1* (0.89 and 0.97, *P* < 0.01) at the same condition (Figure 8). Furthermore, a significant correlation (*r* = 0.95^∗∗^) was detected between expression levels of *AVP1* and *NHX1* genes at the same condition (Figure 8). The expression levels of *AtPAP17* and *AtPAP17* genes were closely associated with the high expression level of salt overly sensitive (SOS) pathway genes and the maintenance of a low Na^+^ and high K^+^ concentrations and, consequently, a high K^+^/Na^+^ ratio (Figure 9).

**FIGURE 10:**
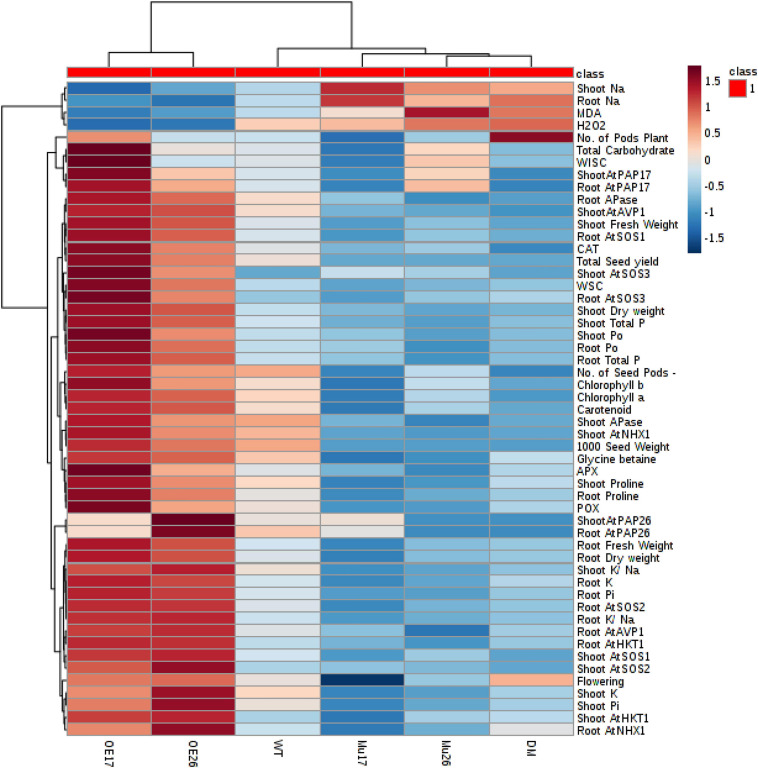
A heat map of the impact of overexpression and knockout of *AtPAP17* and *AtPAP26* genes on the molecular, physiological, biochemical, and morphological parameters of the studied genotypes at 150 mM NaCl stress. Wild-type (WT), overexpress (OE), single mutant (Mu), and double mutant (DM) genotypes of *Arabidopsis thaliana* purple acid phosphatase 17 (*AtPAP17*) and 26 (*AtPAP26*) genes. The color key represents the quantile-normalized log10-transformed values. Dark red indicates high level whereas dark blue indicates low level.

**FIGURE 11:**
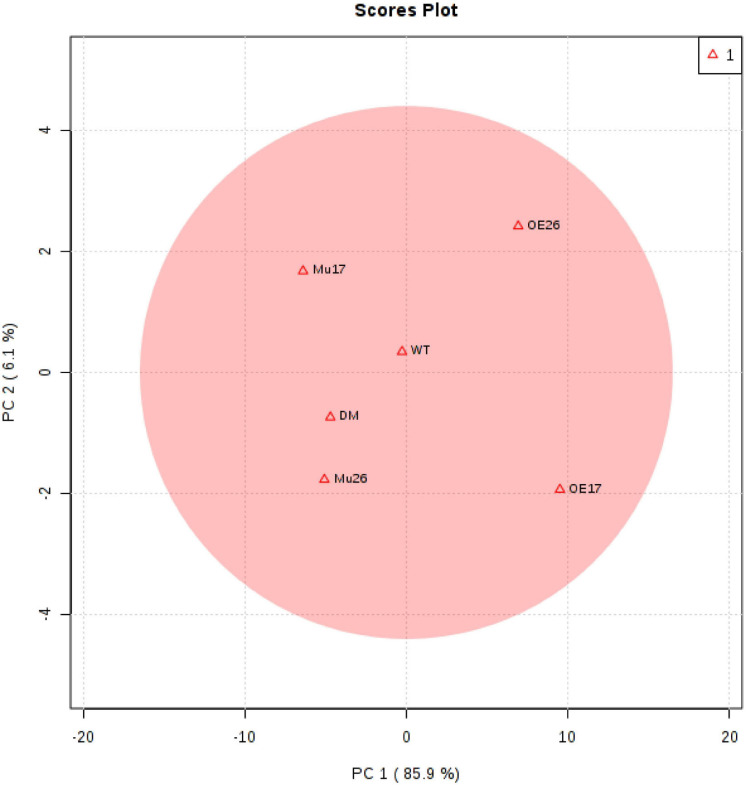
Principal component analysis (PCA) results for all morphological and physiological parameters of the examined genotypes to show grouping of them 150 mM NaCl stress. Wild-type (WT), overexpress (OE), single mutant (Mu), and double mutant (DM) genotypes of *Arabidopsis thaliana* purple acid phosphatase 17 (*AtPAP17*) and 26 (*AtPAP26*) genes.

To clarify a global overview of the effects of AtPAP17 and AtPAP26, a clustered heat map of Pearson’s correlation was performed separately for all measured parameters and each genotype at normal condition and the high level of NaCl concentration as well (Figure 10). The cluster analysis showed obviously that OE17 and OE26 genotypes responded strongly to a higher level of the traits responsible for the salt-tolerance improvement and better growth, compared to WT plants. However, a lower value of these traits was observed in mutant genotypes as compared with WT ones (Figure 10). Since the genotypes presented the different effects on all measured traits, PCA with the full set of traits was performed to further assess the effect of AtPAP17 and AtPAP26 (Figure 11). According to the PCA, an obvious separation was detected among genotypes, where WT plants located between OE and mutant genotypes. Both OE17 and OE26 genotypes were clearly clustered separately with positive loading on the right side of WT, whereas the mutant genotypes were located on the left side of WT (Figure 11).

## Discussion

High salinity reduces the agricultural productivity of most crops intensively (Woodrow et al., 2017; Zörb et al., 2019). Salt levels that are harmful to crop growth affect large arable lands of the world (Yamaguchi and Blumwald, 2005). Hence, this makes it inevitable that the effective strategies involving in salt tolerance to alleviate the deleterious influences of salt stress, to maintain the optimum growth and yield.

Low phosphorus availability is a major constraint for photosynthesis and therefore carbohydrate production since restricts the RuBP regeneration, activation of Rubisco and other PCR cycle enzymes (such as FBPase and SBPase), carbon assimilation, and carbon partitioning (Rychter and Rao, 2005). For example, phosphorus is also one of the necessary components in the sucrose synthesis pathway (Rychter and Rao, 2005). In response, maintaining the homeostasis of phosphate is one of the well particular roles of PAPs (Del Pozo et al., 1999; Veljanovski et al., 2006; Plaxton and Tran, 2011). Therefore, the OE genotypes with more APase activity could further mediate the phosphate homeostasis, which resulted in enhancing the carbohydrate content and the related trends in salt stress conditions.

Plant physiological responses to water deficit stress induce osmotic adjustment to maintain current water uptake and cell turgor (Chaves et al., 2009). OE genotypes were more effective in the enhancement of proline and glycine betaine accumulation in plants under high concentrations of NaCl, which can increase osmotic pressure, and mutant genotypes were the least effective plants in this way. Although glycine betaine and proline are kinds of well-studied compatible solutes, the effect of phosphorus on their production is still unknown. Despite this, according to pathways of proline and synthesis (Lehmann et al., 2010; Meena et al., 2019), which depend on phosphate, we hypothesize that there is a relationship between phosphate supply and the metabolic pathway. Several studies showed that quite a few factors could play multifunctional roles in adaptation to salt tolerance with increasing the activity of antioxidant enzymes, the accumulation of proline and glycine betaine, and so on. For example, supplementation of acetylcholine (ACh), jasmonic acid, nitric oxide, and salicylic acid ameliorated the negative influences of salt stress on plant growth (Ahanger and Agarwal, 2017; Ahmad et al., 2018a, b; Ahanger et al., 2020; Qin et al., 2020).

Oxidative stress, as a secondary effect of salt stress, causes damage to macromolecules such as lipid, protein, and DNA. Plant cells respond to oxidative stress by increasing enzymatic and non-enzymatic antioxidant activity (Ahmad et al., 2019). Since the *AtPAP17* and *AtPAP26* genes via enzymatic antioxidant activity involve in the metabolism of ROS (Del Pozo et al., 1999; Veljanovski et al., 2006), overexpression of which led to the highest enzymatic antioxidant activity, and consequently, the lowest amount of H_2_O_2_ as well as MDA in OE17 and OE26 plants, under high-salinity treatment (150 mM NaCl) (Table 3 and Figure 7). Conversely, the knockout mutation of these genes resulted in the lowest enzymatic antioxidant activity and, thus, the most amount of H_2_O_2_ and MDA amount in mutant plants at the same conditions (Table 3 and Figure 7). Numerous studies have shown a correlation between up-regulation of specific enzymatic antioxidant activities and tolerance to abiotic stresses, including salinity (Bartels and Sunkar, 2005; Hanin et al., 2016; Ahmad et al., 2019). Therefore, the raise of the enzymatic antioxidant activities in OE genotypes positively correlated with improving the parameters affecting the plant growth. Improving the management of ROS and decreasing cell damages in OE17 and OE26 plants could be referenced as an important biological basis for better plant growth features, as well as increasing the biomass, yield components, and total yield, under salt conditions (Figure 10).

Regarding the acid phosphatase (APase) functions, having perceived salinity signal by plant cells, the ultimate biochemical and molecular responses depend on the signaling molecules involved in the activation of signaling pathways, which are able to enhance plant ability to salinity tolerance (Hanin et al., 2016; Isayenkov and Maathuis, 2019; Zhao et al., 2020). Protein phosphatases and protein kinases that catalyze reversible phosphorylation are of utmost importance in signal transduction and regulating metabolic activities as well. Indeed, phosphatases, as the obligate partners of kinases, are half of a phosphorylation/dephosphorylation switch, which have a critical role in the signaling pathways (DeLong, 2006). The regulation of ABA signaling, for example, in plant guard cells is mediated by a protein kinase-phosphatase pair, which interacts with an ion channel to regulate stomatal movements (Lee et al., 2009). To give another example, a Ca^2+^/calmodulin-dependent protein phosphatase mediates a salt-stress signal transduction pathway that influences salt tolerance via the regulation of sodium influx and efflux (Pardo et al., 1998).

Considerably, the maintenance of low Na^+^ and high K^+^ concentrations in plant tissues is the most effective strategy to tolerate high levels of salinity. In fact, the high K^+^/Na^+^ ratio is very important for many species to maintain a low concentration of Na^+^ (Tester and Davenport, 2003; Deinlein et al., 2014). Also in the current study, the correlation between K^+^/Na^+^ ratio and dry weight of the seedling (0.94) was strongly higher than the correlation between Na^+^ content and seedling dry weight (0.82). the pieces of evidence show that K^+^ ion activates more than 50 enzymes and requires for protein synthesis as well as many physiological and biological processes, while Na^+^ ion cannot substitute for this role (Tester and Davenport, 2003; Isayenkov and Maathuis, 2019). Metabolic toxicity of Na^+^ is drastically a result of its ability to compete with K^+^ for the occupation of binding sites essential for cellular functions (Tester and Davenport, 2003). Thus, the high level of Na^+^ or low K^+^/Na^+^ ratios more likely could disrupt various enzymatic processes in Mu17, Mu26, and DM cytoplasm. These results were supported by the overexpression of *PAP17* and *PAP26* that caused the low sensitivity in OE17 and OE26 plants with high salt stress (Figure 10). The alteration of the biomass, yield components, and total yield among genotypes studied (at 150 mM NaCl) obviously confirmed that the K^+^/Na^+^ balance will be the downstream effect of *PAP17* and *PAP26* in high salt stress (Figure 10).

The SOS pathway has a critical role in establishing ion homeostasis, and consequently, plant adaptation to salt stress (Ji et al., 2013). At the beginning of this pathway, SOS3, a myristoylated calcium-binding protein, senses increasing in salinity-induced cytosolic Ca^2+^. For more explanation, the concentration of Ca^2+^ and inositol 1,4,5-trisphosphate (IP3) in the cytosol increased by NaCl stress. The increase in IP3 concentration, which hydrolyzes through phosphatidylinositol 4,5-bisphosphate (PIP2), also mediates cellular Ca^2+^ mobilization. The translocation of cytosolic calcium mediates through Ca^2+^ channels and pumps depending on ATPase activity, which are presented on the membranes (Park et al., 2016). According to our results, the enhanced capacity of APase activity and phosphate availability in overexpress genotypes can improve the influx and release Ca^2+^, and consequently enhance *SOS3* expression in OE17 and OE26 plants at high salt stress. The relationship between the Pi content and *SOS3* expression level strongly supported this conclusion (Figure 8). Then, the activated SOS3 protein physically interacts with the auto-inhibitory domain of SOS2, which form the SOS2/SOS3 complex. The kinase complex phosphorylates and regulates other genes: *NHX1*, *AVP1*, and *SOS1*, to prevent the excess Na^+^ accumulation in the plant cymplast (Hanin et al., 2016; Isayenkov and Maathuis, 2019; Zhao et al., 2020). Due to the nature of serine/threonine protein kinase SOS2, the activity of which dependents on phosphate concentration intensively. Here, a significant positive (*P* < 0.05) correlation was observed between the expression level *SOS2* and SOS3 as well as the Pi content and *SOS2* expression level at 150 mM NaCl (Figure 8). Therefore, OE17 and OE26 genotypes with a better ability of phosphate homeostasis in plant cells exhibited significantly higher *SOS2* expression.

Sodium compartmentation into vacuoles is another plant strategy for maintaining the lower ion concentration and minimizing the deleterious effects of excess Na^+^ in the cytosol. The NHX1, as a vacuolar Na^+^/H^+^ transporter, plays a critical role in sequestering Na^+^ into the vacuole (Liang et al., 2015; Park et al., 2016). Hence, genotypes with higher expression and activity of NHX1 would be more successful in stress conditions. Here, the highest *NHX1* expression level belonged to the overexpressed genotypes, both in shoot and root tissues of OE17, and shoot of OE26 genotype. Since SOS2/SOS3 kinase complex phosphorylates and activates the vacuolar Na^+^/H^+^ transporter in the cells, the enhanced *SOS2* expression levels in the overexpressed genotypes led to an increase in *NHX1* expression level (Figure 8). The inverse results were obviously observed for the mutant genotypes, as *NHX1* expression decreased significantly in the shoots of three mutant genotypes and Mu26 roots compared to that in WT ones (Figure 4).

The vacuolar Na^+^/H^+^ antiporter is driven by an electrochemical gradient of protons across the tonoplast that can be generated by vacuolar H^+^-pumps, H^+^-ATPase, or H^+^-pyrophosphatase (H^+^-PPase) type. AVP1 is a vacuolar H^+^-PPase that acidifies vacuoles in plant cells (Maeshima, 2000; Zhao et al., 2020). The results showed that OE17 and OE26 plants with higher levels of *SOS2* and *SOS3* expression and more PPi availability in the plant cells could be likely more efficient in enhanced activity and expression of *AVP1* at high salt stress (Figure 9 and Table 4). We also suggest that the increased *AVP1* expression level in OE17 and OE26 plants provided an additional driving force for vacuolar sodium accumulation via the vacuolar Na^+^/H^+^ antiporter, NHX1 (Figures 8, 9). These results were also confirmed by mutant genotypes, in which the *AVP1* expression level significantly decreased both in shoot and root tissues of all three mutant genotypes compared to WT plants at the same condition. Further, this is obviously verified by the correlation between the *NHX1* expression level and *SOS3* and *SOS2* expressions as well as the relationship between the Pi content and *NHX1* expression level (Figure 8). Besides, the compartmentation of Na^+^ could maintain the turgor of transgenic plants, since increase in cellular solute content might lead to an increase in the uptake of water (Gaxiola et al., 2001). Our findings of changes in the proline and glycine betaine potential in the shoots and roots of genotypes studied at high salt stress condition (Table 3) are consistent with this idea.

SOS2/SOS3 kinase complex also associates with SOS1 transporter phosphorylation in the plasma membrane and activating Na^+^ efflux from the cytosol to apoplast (Katiyar-Agarwal et al., 2006; Ji et al., 2013). This antiporter is powered by the operation of the plasma membrane H^+^-ATPase that definitely associates with available phosphate. Therefore, according to these pieces of evidence and more APase activity of OE genotypes, we propose that the rising in SOS2/SOS3 kinase complex activity and plasma membrane H^+^-ATPase was desirable for increasing antiporter SOS1 activity in OE17 and OE26 genotypes. The result provides a feasible way to improve *SOS1* expression level. Shi et al. (2000) have reported that the expression level of *SOS1* in plants is up-regulated in response to NaCl stress and this up-regulation is also abated in SOS3 or SOS2 mutant plants. The relationship between *SOS1* expression and the *SOS3* and *SOS2* expression levels and the Pi content confirmed the mentioned results (Figure 8).

To deal with Na^+^-specific toxicity in the cytosol, plant cells also restrict Na^+^ influx. The *HKT1* gene is a selective Na^+^ transporter that mediates K^+^ transport as well. In fact, HKT1-type transporters are responsible for the balance between Na^+^ and K^+^ ions under salinity stress (Yokoi et al., 2002; Ali et al., 2019). Increased levels of *HKT1* gene expression in OE plants might indicate increased Na^+^ uptake into the cells. However, other roles of HKT1 in various tissues can shed more light on this hurried conclusion including the Na^+^ loading regulation into the root xylem, Na^+^ loading into the phloem sap in shoot, and limiting Na^+^ influx to root (Chinnusamy et al., 2006; Munns and Tester, 2008; Hasegawa, 2013; Ali et al., 2019). The high K^+^/Na^+^ ratio in OE17 and OE26 plants provided evidence for the hypothesis that up-regulation of *HKT1* gene led to the regulation of Na^+^ and K^+^ homeostasis under salinity stress. maintaining a high K^+^/Na^+^ ratio in the cytosol is critical for the function of cells, especially under high-salinity condition (Deinlein et al., 2014; Zhao et al., 2020).

The high expression level of SOS pathway genes and their relationship with the maintenance of a high K^+^/Na^+^ rate could be considered for future salinity tolerance in OE genotypes (Figure 9). In addition, maintaining a high K^+^/Na^+^ ratio could have a relevant role in conferring tolerance to stress combination, both as a low destructive effect on plant cells and as a regulatory effect in the protection of cell lipid, proteins, and DNA damages from oxidative processes. The changes observed in H_2_O_2_ and MDA content of the leaves in different genotypes (Table 3) can also demonstrate the interaction between SOS1 and RCD1 (radical-induced cell death), which is a transcriptional regulator of ROS homeostasis (Katiyar-Agarwal et al., 2006).

All these properties of OE17 and OE26 plants can be helpful in establishing lower Na^+^ concentration in the cytosol (Figure 9). This situation largely accomplished through *PAP17* and *PAP26* roles in more cytosolic Na^+^ transportation into the vacuole and apoplast space, consequently, alleviating the Na^+^ toxic effects. These results were closely associated with the photosynthetic pigments and total carbohydrate content, hence, the higher photosynthesis capacity indicated in OE17 and OE26 compared to those in WT plants (Table 2). These conclusions were supported by investigation of the studied characteristics in Mu17, Mu26, and DM plants. Therefore, these results can introduce such an important and basic strategy to improve the growth and yield features of plants, since the living cells need strategies to protect them from the Na^+^ ion damage, under salt stress condition (Xu et al., 2011).

Concerning plants’ phosphate, the results revealed that the salt stress decreases the phosphate content and increases the APase activity in plants, which were in agreement with previous reports (Martinez and Lächli, 1991; Martinez and Läuchli, 1994; Navarro et al., 2001; Parida and Das, 2005; Brown et al., 2006) who reported the acquisition and utilization of Pi are decreased in plants under salt stress. Induction of APase activity is one of the essential indicators of plant response to Pi starvation (Abel et al., 2002; Yuan and Liu, 2008; Tran et al., 2010a). The expression of both *PAP17* and *PAP26* genes also was strongly induced by high salt stress (150 mM NaCl), and the expression levels closely correlated with the APase activity (Figure 6). These results are in agreement with previous studies, documenting the constitutive expression of *PAP17* and *PAP26* transcripts under high salt stress in *Arabidopsis* (Del Pozo et al., 1999; Lohrasebi et al., 2007).

At high salt stress, overexpression of *PAP17* and *PAP26* could result in increasing intracellular APase activity in both OE genotypes, where exhibited significant increases in the total P content, Pi, and Po content as compared to WT plants. These results were closely associated with the improved physiological, biochemical, and molecular responses to boost salt tolerance (Figures 10, 11). Improving all these characteristics would be beneficial for higher photosynthesis capacity and, ultimately, more efficient in growth of OE17 and OE26 plants (Figures 10, 11). Our measurements of the studied characteristics are supported by loss of *PAP17* and *PAP26* function in mutant genotypes (Mu17, Mu26, and DM) that showed sensitivity to high salt stress (Figures 10, 11). Several studies have reported that overexpression of secreted PAPs can improve P accumulation and plant biomass (Xiao et al., 2006; Hur et al., 2007; Ma et al., 2009; Wang et al., 2009).

The extra Pi, which was released and accumulated in the cell by overexertion of AtPAP26, may be the possible reason that more significant APase activity was not detected in OE26 shoots compared to WT shoots at 150 mM NaCl. Previous studies had indicated that *AtPAP26* is a major intracellular and secreted APase as well (Veljanovski et al., 2006; Hurley et al., 2010; Tran et al., 2010b). This hypothesis is confirmed by comparing the Pi content of shoots between OE26 and WT plants, even comparing with OE17 shoots at 150 mM NaCl. Indeed, Pi content of OE26 shoots was 1.03-fold, and 0.33-fold more compared to that in WT and OE17 shoots, respectively, at the same condition (Table 4).

At first glance, it is difficult to believe that MU26 or DM plants grown at 0 or 50 mM NaCl presented a similar or greater shoot APase activity as compared with that in WT plants. Nonetheless, similar results have been obtained by Veljanovski et al. (2006); Hurley et al. (2010), and Farhadi et al. (2020) who reported that the elimination of a member of the AtPAP family could simultaneously exert a significant stimulating effect on nonspecific APase activity and growth of lack-Pi Arabidopsis. Elimination of the AtPAP17 and AtPAP26 functions, in fact, positively influences the expression and activity of other APases–particularly AtPAP26 in *atpap17* mutant and AtPAP17 in *atpap26* mutant (Rouached et al., 2010; Farhadi et al., 2020) and/or Pi transporters. In other words, this confirms the recovery and compensation roles of AtPAP17 and AtPAP26 under phosphate deficient conditions.

Our results showed that knockout mutation of AtPAP17 or AtPAP26 genes led extremely to sense the phosphorus imbalance in Mu17, Mu26, and DM plants imposed to salinity and even normal conditions (0 mM NaCl) compared to wild-type and OE lines at the same condition. It seems that phosphate homeostasis in cells of mutant plants was also disturbed at normal condition (0 mM NaCl) since the functions of these genes can be attributed to the dual role of the main components of the compensatory network (Hurley et al., 2010; Farhadi et al., 2020). In other words, the activity of the compensatory network in response to these two genes’ destruction could not entirely compensate the intercellular Pi homeostasis at normal condition. Several studies have also indicated that the absence of AtPAP12, AtPAP17, and AtPAP26 was compensated by upregulation of other PSI PAP isozymes (Tran et al., 2010b; Robinson et al., 2012; Farhadi et al., 2020). However, when the functions of AtPAP17 and AtPAP26 were eliminated in the Mu17, Mu26, and DM plants, their functions could not be fully compensated by other PSI PAP isozymes at 100 and 150 mM NaCl, which led to a more severe phosphate deficiency (Table 4).

To sum up, our results showed that nonspecific APases most likely had compensation roles in mutant plants to enhance Pi releasing, recycling, and scavenging from both internal and external resources. However, with increasing NaCl concentration from 50 to 100 and 150 mM, these compensation roles were not showed in mutant plants (Table 4).

These results indicated that apparently both *PAP17* and *PAP26* genes are involved in plant response to salt tolerance by controlling various downstream biological pathways. The stimulus of these both genes could be derived from the critical roles of their APase activity and non-APase activity, such as alkaline peroxidase activity (Figure 7). This conclusion was supported by comparing the *PAP17* expression level in OE17 shoots and roots, and the *PAP26* level expression in OE26 shoots and roots at 0 and 150 mM NaCl. It is herewith suggested that the non-APase activity role of these two is confirmed by the increased expression in *PAP17* and *PAP26* in OE17 and OE17 genotypes at high salinity, respectively, since there were adequate levels of the gene expression for Pi homeostasis.

## Conclusion

Overall, to our knowledge, this study is the first report describing the effects of *AtPAP17* and *AtPAP26* genes in salt stress conditions. This could open a new insight of salt tolerance in *A. thaliana* with improvement in various aspects of the molecular, biochemical, physiological, and morphological mechanisms. The results clearly pointed out that AtPAP17 and AtPAP26 proteins are responsible as two novel regulators engaged in salt tolerance. It is also highlighted the fact that *AtPAP17* and *AtPAP26* genes would enable plants to activate the numerous biochemical pathways toward adaptive responses to high-stress salt. This could be such a direct and/or indirect influence that minimizes the deleterious effects of the stress. Providing such a novel practical and approach to boost salt tolerance is the main point that distinguishes this study from previous ones. In fact, the genetic modification of such genes that motivate a group of genes involved in salt tolerance might be the most appropriate strategy for salt tolerance since many genes determine plant adaptation. Consequently, we suggest *AtPAP17* and *AtPAP26* genes, as two candidate genes, for molecular breeding of salt-tolerance enhancement in crop plants.

## Data Availability Statement

The original contributions presented in the study are included in the article/supplementary material, further inquiries can be directed to the corresponding author/s.

## Author Contributions

MA-V performed all the experiments and data analysis, and wrote the manuscript. MS supervised the project and provided editorial input into the writing. GK advised the project. All authors contributed to the article and approved the submitted version.

## Conflict of Interest

The authors declare that the research was conducted in the absence of any commercial or financial relationships that could be construed as a potential conflict of interest.
